# Comparative genomics of *Pseudomonas syringae* pv. *syringae* strains B301D and HS191 and insights into intrapathovar traits associated with plant pathogenesis

**DOI:** 10.1002/mbo3.261

**Published:** 2015-05-04

**Authors:** Aravind Ravindran, Neha Jalan, Joshua S Yuan, Nian Wang, Dennis C Gross

**Affiliations:** 1 Department of Plant Pathology and Microbiology, Texas A&M UniversityCollege Station, Texas, 77843-2132; 2 Department of Microbiology and Cell Sciences, Citrus Research and Education Center, University of Florida700 Experiment Station Road, Lake Alfred, Florida, 33850

**Keywords:** Comparative genomics, effector gene, genome sequence, plant pathogen, *Pseudomonas syringae*

## Abstract

*Pseudomonas syringae* pv. *syringae* is a common plant-associated bacterium that causes diseases of both monocot and dicot plants worldwide. To help delineate traits critical to adaptation and survival in the plant environment, we generated complete genome sequences of *P. syringae* pv. *syringae* strains B301D and HS191, which represent dicot and monocot strains with distinct host specificities. Intrapathovar comparisons of the B301D (6.09 Mb) and HS191 (5.95 Mb plus a 52 kb pCG131 plasmid) genomes to the previously sequenced B728a genome demonstrated that the shared genes encompass about 83% of each genome, and include genes for siderophore biosynthesis, osmotolerance, and extracellular polysaccharide production. Between 7% and 12% of the genes are unique among the genomes, and most of the unique gene regions carry transposons, phage elements, or IS elements associated with horizontal gene transfer. Differences are observed in the type III effector composition for the three strains that likely influences host range. The HS191 genome had the largest number at 25 of effector genes, and seven effector genes are specific to this monocot strain. Toxin production is another major trait associated with virulence of *P. syringae* pv. *syringae*, and HS191 is distinguished by genes for production of syringopeptin SP25 and mangotoxin.

## Introduction

*Pseudomonas syringae* is a Gram-negative facultative phytopathogenic bacterium that is genetically diverse and associated with a variety of plants, both monocots and dicots, grown in native habitats across the world. *Pseudomonas syringae* strains have been isolated from over 180 host plants spanning the plant kingdom, including agriculturally important crops (Hirano and Upper 1990; Young 2010). The first strain of this species was isolated in 1902 from lilac (*Syringa vulgaris*), from which the species name is derived (Young 2010). The species *P. syringae* is subdivided into more than 50 pathovars that were erected to classify phenotypically indistinguishable strain populations with specific pathogenic capabilities toward one or more host plant species. Eventually, nine distinct genomospecies of *P. syringae* were identified by Gardan et al. (1999) based on DNA–DNA relatedness. With the advent of genetic analysis, the genomospecies were shown to be correlated with phylogenetic groups based on multilocus sequence typing (MLST) of conserved housekeeping genes (Bull et al. 2011). Genomospecies 1, which is centered on *P. syringae* pv. *syringae* and related pathovars, also contains nonpathogenic environmental strains that are commonly found in leaf litter in forests, clouds, and various bodies of water (Morris et al. 2013; Vinatzer and Monteil 2014). Nevertheless, it is widely recognized that *P. syringae* pv. *syringae* and related plant pathogenic strains within genomospecies 1 are common epiphytes that maintain resident populations in the absence of plant disease (Hirano and Upper 1990).

The availability of pyrosequencing technologies has greatly expanded the inventory of draft genome sequences of *P. syringae* strains over the past 10 years. Presently, draft genome sequences are publicly available for more than 50 strains of *P. syringae* that include strains from at least 25 designated pathovars (Baltrus et al. 2011; Studholme 2011). This is a testament to the importance of *P. syringae* as both a model pathogen used to decipher the mechanisms of plant pathogenicity and its economic importance as a pathogen distributed worldwide. Yet the complete genome sequences are available at this time for only three strains of *P. syringae* representing three evolutionarily distinct genomospecies. *Pseudomonas syringae* pv. *tomato* DC3000, which is a prominent model strain for studies of plant–bacterial interactions in *Arabidopsis* and other plants, was the first genome completed (Buell et al. 2003) and is representative of genomospecies 3 (Gardan et al. 1999). Subsequently, complete genome sequences were reported for genomospecies 2 strain 1448A of *P. syringae* pv. *phaseolicola* (Joardar et al. 2005), which causes halo blight of bean, and for genomospecies 1 strain B728a of *P. syringae* pv. *syringae* (Feil et al. 2005), which causes brown spot of bean. It is unfortunate with the extensive availability of draft genomes of *P. syringae* strains that the more labor- and time-intensive task of finishing genomes has been skipped because definitive information about genome structure, gene prediction, and gene regulation may be missing, which are critical to revealing novel biological insights (Studholme 2011; Baltrus et al. 2014). This is especially true for comparative genomic analysis of strains within a given *P. syringae* pathovar where they share a conserved set of core genes essential to basic housekeeping functions along with traits important to survival in the plant environment. Because the genomes of *P. syringae* are highly dynamic and driven by horizontal gene transfer processes (Baltrus et al. 2014), comparisons of finished intrapathovar genomes can provide a refined evolutionary perspective on traits vital to plant pathogenicity.

Genomic analyses of *P. syringae* strains within distinct genomospecies revealed that there was limited recombination between phylogenetic groups (Sarkar and Guttman 2004) despite substantial recombination within groups (Cai et al. 2011). Accordingly, Vinatzer and Monteil (2014) proposed that genomospecies of *P. syringae* represent separate recombining populations of crop and environmental strains. This is perhaps best exemplified by *P. syringae* pv. *syringae* classified within genomospecies 1, which is distinguished by a capacity to maintain large epiphytic populations in the absence of disease and to reside within diverse environmental reservoirs (Morris et al. 2013; Vinatzer and Monteil 2014). Characteristically, strains of pathovar *syringae* produce multiple toxins including the syringomycin and syringopeptin lipodepsipeptide toxins (Bender et al. 1999) along with syringolin (Misas-Villamil et al. 2013). Production of these toxins or their synthetase genes have been used repeatedly for identification of pathovar *syringae* and phylogenetically aligned pathovars (Quigley and Gross 1994; Sorensen et al. 1998; Young 2010). In addition, analysis of draft genomic sequences of multiple strains of pathovar *syringae* showed that they carry a smaller inventory of known type III effectors as compared to pathovars classified within other genomospecies (Baltrus et al. 2014). These observations led to the theory that *P. syringae* strains within genomospecies 1 compensate for a reduced repertoire of type III effectors by producing multiple toxins and other virulence factors (Baltrus et al. 2011).

We report the complete genome sequences of *P. syringae* pv. *syringae* strains B301D and HS191, which are phylogenetically related to the B728a genome in genomospecies 1 (Feil et al. 2005). Recently, a draft genome sequence for strain B301D-R was reported (Dudnik and Dudler 2014a). However, the draft B301D-R genome sequence is dispersed among 81 contigs and 21 of these contigs are less than 10 kb in size with many incomplete gene sequences; thus it is not as valuable as our mapped and annotated B301D genome. By conducting comparative genomic analysis relative to strain B728a, our goal was to provide insight into evolutionary processes that are important to plant pathogenesis within a single *P. syringae* pathovar. Strains B301D, a dicot strain, and HS191, a monocot strain, were chosen for sequencing because of apparent differences in host specificity (Gross and DeVay 1977). We also aimed to compare variation within the repertoire of factors associated with virulence including, secretion systems, type III effectors, and toxin production. Ultimately, conserved components of the *P. syringae* core genome will be revealed along with accessory genes unique to individual strains that may be relevant to their distinct lifestyles.

## Experimental Procedures

### Bacterial strains, cultivation, and plant pathogenicity assays

*Pseudomonas syringae* pv. *syringae* strain B301D was originally isolated in 1959 from a diseased flower of the common pear (*Pyrus communis*) variety “Comice” by C. M. E. Garrett at the East Malling Research Station, Maidstone, Kent, England. The strain was originally designated as strain #55 prior to its transfer to a collection in California as strain B301D (Gross and DeVay 1977). Strain HS191 of *P. syringae* pv. *syringae* was originally isolated in Australia by A. C. Hayward from diseased proso millet (*Panicum miliaceum*) in October 1969. The strain is deposited in other collections under several accession numbers (B497, NCPPB 1242, Dye P192, CFBP 3318, ICMP 4860, LMG 5647, PDDCC 3024, and 5D430). The 5D430 accession (Gross and DeVay 1977) was renamed as HS191 (Gonzalez et al. 1984) and is also listed as B359 (Ballio et al. 1991). *Pseudomonas syringae* pv. *syringae* B728a (Feil et al. 2005) was used as a reference strain for pathogenicity tests and plant host range studies. The bacteria were grown routinely at 25°C in nutrient broth-yeast extract (NBY) liquid or on NBY agar medium (Vidaver 1967). Strains were routinely stored in glycerol in at −80°C, and were preserved long-term as lyophilized reference stocks.

Strains B301D, HS191 and B728a were evaluated for ability to cause disease symptoms in bean plants (*Phaseolus vulgaris* “Blue Lake 274”) and tobacco (*Nicotiana benthamiana*) using methods previously described (Greenwald et al. 2012). Bacterial suspensions at high (5 × 10^8^ colony-forming units [CFU]/mL) and low (5 × 10^6^ CFU/mL) were used to inoculate 2-week-old bean and 4-week-old tobacco plants by vacuum infiltration. Three plants were inoculated per strain, and pathogenicity tests were repeated on two occasions. Control plants were infiltrated with sterile distilled water. Disease symptoms were monitored over a 5-day period following inoculation.

For tests of copper resistance, strains B728a, B301D and HS191 were streaked in mannitol glutamate-yeast extract (MGY) agar (Keane et al. 1970; Bender and Cooksey 1986) incorporated with CuSO_4_ at different concentrations (0.4, 0.8, 1.6, and 3.2 mmol/L). Log phase cultures of the strains were diluted to 10^6^ CFU/mL in sterile water, spotted (10 *μ*L) on MGY media with presence and absence of CuSO_4_, and incubated at 28°C for 3 days (Bender and Cooksey 1986). Formation of bacterial colonies was used to determine the end point sensitivity to copper.

Assays for ice nucleation activity were performed by the method of (Lindow et al. 1978). Strains B728a, B301D and HS191 were grown on NBY agar for 4 days, cells were suspended in 0.5 mL of distilled water (∼10^8^ CFU/mL). Bacterial suspensions (100 *μ*L) adjusted to 10^8^, 10^7^, 10^6^, and 10^5^ CFU/mL in microfuge tubes were tested for ice nucleation activity in a −5°C ethanol-water solution in bath for 3 min. Tubes (three replicates each) with ice nucleation activity were observed and recorded.

### DNA isolation

Genomic DNA was extracted from bacterial cultures of strains B301D and HS191 grown to log-phase at 28°C in NBY liquid medium. High quality genomic DNA was extracted from cells washed in Tris-EDTA buffer using a cetyl trimethyl ammonium bromide (CTAB) protocol recommended by the DOE Joint Genome Institute (JGI) for isolation of genomic DNA from bacteria (http://my.jgi.doe.gov/general/protocols.html). A spectrophotometer (Nanodrop ND-1000; NanoDrop Tech. Inc., Wilmington, DE) was used to measure the DNA quantity and ensure the DNA quality was suitable for sequencing.

### Genome sequencing and assembly

For both strains B301D and HS191 between 4 and 5 *μ*g of DNA was isolated and used for two high-throughput DNA sequencing methods. First, 454 pyrosequencing (http://www.454.com/) of single and paired-end reads of genomic DNA fragments were generated on a 454 GS-FLX Titanium sequencer (454 Life Sciences, Branford, CT) at the Interdisciplinary Center for Biotechnology Research, University of Florida (Jalan et al. 2011). Subsequently, Illumina sequencing (http://www.illumina.com/) generated single and paired-end sequence reads of genomic DNA, and was conducted using an Illumina Genome Analyzer IIx (Illumina, Hayward, CA) at the Genomics and Bioinformatics Services of Texas A&M AgriLife Research, College Station, TX, USA.

The 454 sequencing of B301D and HS191 genomic DNA, respectively, yielded total reads of 451,081 and 367,109 with an average length of 300 bp. High-quality sequences were assembled into contigs and scaffolds using the 454 de novo assembler Newbler 2.0. A total of 363 contigs averaging 20 kb in size were generated for B301D, of which 302 contigs were larger than 500 bp. The B301D contigs were further grouped into twelve scaffolds based on paired-end reads. A total of 363 contigs averaging 16 kb in size were generated for HS191, of which 311 contigs were larger than 500 bp. The HS191 contigs were then grouped into seven scaffolds based on paired-end reads, with one scaffold at 52,548 bp representing plasmid pCG131 (Gonzalez et al. 1984). Illumina sequencing of B301D and HS191 genomic DNA, respectively, generated a total of 19,110,198 and 63,331,996 high-quality filtered sequence reads with an average length of 76 bp. The average coverage of the genome for both strains was more than 400-fold. Illumina sequence yielded 2292 and 1287 contigs, respectively, for strains B301D and HS191. Subsequently, the Illumina contigs were aligned against the 454 scaffolds by using BLASTn to confirm the orientations and integrity of the assembled sequences and to close gaps and link contigs together within the scaffold. CLCbio Genomics Workbench version 4.9 (Boston, MA, USA) was used for de novo assembly of reads; all other parameters were set at default values except for similarity set at 0.9.

After extensive efforts to generate high quality genomic sequences, the assembly of genomes generated 12 and six scaffolds, respectively, for strains B301D and HS191, with internal gaps due to repeat regions. Optical mapping then was used to obtain a de novo KpnI restriction map of each strain’s genome with no prerequisite for sequence information (Latreille et al. 2007). A de novo KpnI optical map of the B301D and HS191 genomes was generated by OpGen Technologies (Madison, WI). The DNA scaffolds generated by Newbler assembly (12 scaffolds, B301D; six scaffolds, HS191) were then aligned to the optical map according to their KpnI restriction fragment pattern using MapSolver v.3.1 software (OpGen Technologies, Inc.) (Jalan et al. 2011) (Fig. S7). Sequence gaps between the scaffolds were identified and PCR primers were designed for gap closure. The genomes were completely closed by primer walking and validated by manually inspecting all areas of imperfect match between the optical map and the sequence assembly. Standard PCR procedures using Phusion® high fidelity DNA polymerase (Thermo Fisher Scientific Inc., Waltham, MA, USA) were used, and then the PCR products were subjected to traditional Sanger sequencing. Gaps were closed by correlating KpnI sites within the amplified DNA sequence with the optical map generated by OpGen to verify continuity of DNA sequence. The genome sequences were further validated either by high coverage with 454 and Illumina sequence data or by resequencing of the region (data not shown).

### Annotation, curation, and features of genomes

Once the corrected genome sequences for B301D and HS191 were confirmed by optical scanning, they were submitted to the Integrated Microbial Genomes Expert Review (IMG/ER) system for annotation by gene calling (Markowitz et al. 2009). The IMG annotation system predicted functions for the B301D and HS191 genomes based on matches to a combination of functional annotation databases (COG, Pfam, TIGRfam, InterPro, Gene Ontology, and KEGG). The JGI GenePRIMP pipeline (Pati et al. 2010) was used to identify incorrectly annotated genes that were subsequently corrected using Artemis (Rutherford et al. 2000). The finished B301D genome was 6.09 Mb in size, whereas the HS191 genome was 5.94 Mb in size and contained a 52-kb plasmid called pCG131 (Gonzalez et al. 1984). The CGView Server was used to generate graphical views of the genome (Grant and Stothard 2008).

### Phylogenetic analysis

**Figure 1:**
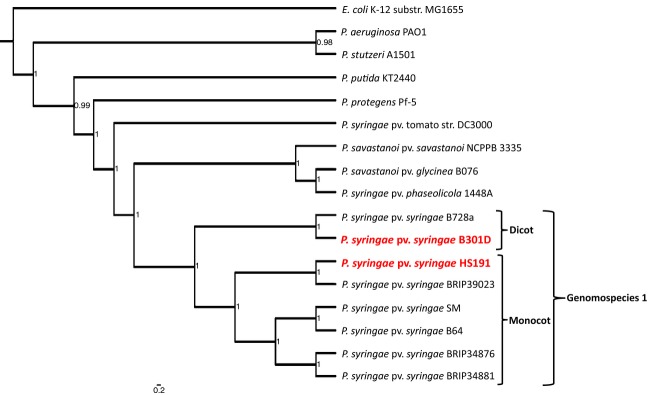
Phylogenetic relationships of the genomes of strains B301D and HS191 to other *Pseudomonas* species and pathovars. The maximum likelihood tree was constructed using concatenated alignments of 10 housekeeping genes: *acsA*, *aroE*, *dnaE*, *guaA*, *gyrB*, *mutL*, *ppsA*, *pyrC*, *recA*, and *rpoB*. The MrBayes package (Ronquist and Huelsenbeck 2003) was used to calculate the clade credibility values shown as values within interior nodes. Strains of *Pseudomonas syringae* in genomospecies 1 are indicated by the outside bracket and the strains originating from monocot and dicot hosts are as indicated by two inside brackets. Strains B301D and HS191 that were sequenced in this study are shown in red, enlarged lettering. The outgroup genome sequence used was *Escherichia coli* K-12 substrain MG1655.

The evolutionary position of *P. syringae* pv. *syringae* strains B301D and HS191 relative to other pathovars and species of *Pseudomonas* was determined by multi-locus sequence analysis (MLSA) using methods described previously (Loper et al. 2012). Several strains selected for phylogenetic analysis were available as draft genome sequences, but they contained the full housekeeping gene sequences necessary for MLSA. Genomospecies 1 was represented by *P. syringae* pv. *syringae* strains SM, B64, BRIP 34876, BRIP 34881, and BRIP 39023 (Gardiner et al. 2013; Dudnik and Dudler 2014b) in addition to B728a (Feil et al. 2005). The other *Pseudomonas* strains used in the analysis are listed in Figure1. The MLSA data is based on phylogenetic analysis of the DNA sequences of 10 housekeeping genes (i.e., *acsA*, *aroE*, *dnaE*, *guaA*, *gyrB*, *mutL*, *ppsA*, *pyrC*, *recA*, and *rpoB*) obtained in this study or available at the NCBI GenBank database. DNA sequences were aligned by concatenation using the MEGA5 program (Tamura et al. 2011) with default settings. Maximum Likelihood analysis was performed for 1000 bootstrap replications using the equally weighted heuristic search option in PAUP v4.0 (Swofford 2002). A general time-reversible model with variable sites and discrete gamma distribution (GTR+I+G) as a best-fit model was selected by Akaike Information Criterion (AIC) in MrModeltest v2.3 (Nylander 2004). Bayesian analyses were run for 3,500,000 generations for housekeeping genes, performed in MrBayes v3.1.2 (Ronquist and Huelsenbeck 2003). Posterior probability (PP) values were subsequently calculated. Every 100th tree after stabilization (burn-in) was sampled to calculate a 50% majority rule consensus tree. The final phylogenetic tree was constructed using the program FigTree v1.3.1 (http://tree.bio.ed.ac.uk/software/figtree/).

### Comparative genome analysis

The genome sequences of *P. syringae* pv. *syringae* strains B301D and HS191 were compared to the genome of strain B728a (GenBank accession no. NC_007005) and its closest relatives in the GenBank database, based on BLAST and phylogenetic analyses. Complete genome sequences of all three *P. syringae* pv. *syringae* strains (B728a, B301D and HS191) were aligned and visualized in progressive mode using MAUVE (Darling et al. 2010). Pangenome analysis that included the shared genes by all the three strains was done by an “all-against-all” BLAST of the protein sequences of the above genomes. The genes aligned based on amino acid sequence were considered orthologous if reciprocal BLASTp hits were found between two genes with *e* values of ≤10^−20^ and alignments exceeding 60% sequence identity and 60% query gene length. A gene was considered a singleton or unique to each strain if it had no hits with an *e* value of ≤10^−5^.

A circular comparison of the genomes of the strains B728a, B301D and HS191 was generated using BRIG analysis (http://brig.sourceforge.net/) (Fig. 3A–C). BRIG, or the BLAST Ring Image Generator (Alikhan et al. 2011; Edwards and Holt 2013) is a Java-based tool for visualizing the comparison of a reference sequence to a set of query sequences. Results were plotted as a series of rings, each representing a query sequence, which is colored according to BLAST identity (100%, 70%, and 50%) to indicate the presence of hits to the reference sequence. BRIG analysis was also used to evaluate the HS191 plasmid (pCG131) as a central reference sequence against pSM1, pPSR1, pPMA4326A, pA506 and the pPph1448A-B plasmid, previously described for strains of *P. syringae* (Joardar et al. 2005; Zhao et al. 2005, Stockwell et al. 2013; Dudnik and Dudler 2013b).

Type III secretion system (T3SS) effectors in strains B301D and HS191 were identified using the protocol described by Baltrus et al. (2011). The list of effector sequences were compiled from the *Pseudomonas* effectors database (http://pseudomonas-syringae.org) together with additional effector sequences (Baltrus et al. 2011; Bart et al. 2012). Effector sequences were systematically used in BLASTn analysis of the B301D and HS191 genomes to identify the complement of effectors specific to each strain. Comparison of genes and operons associated with production of the toxins, syringomycin, syringopeptin, and mangotoxin were compared among the three strains of *P. syringae* pv. *syringae* (B728a, B301D, and HS191) by IMG gene content analysis (Mavromatis et al. 2009). Insertion sequences were identified by submitting the whole genome of a strain to the IS Finder website (Siguier et al. 2006). Finally, the B301D and HS191 genomes were analyzed for phage origins using Prophage Finder (http://bioinformatics.uwp.edu/∼phage/ProphageFinder.php).

### Nucleotide sequence accession number

The complete genome of *P. syringae* pv. *syringae* strain B301D chromosome is deposited at NCBI under the accession number CP005969. The complete genome of strain HS191 and its pCG131 plasmid are deposited under accession numbers CP006256 and CP006257, respectively.

## Results and Discussion

### Pathogenicity tests and host range

Strains B301D and HS191 of *P. syringae* pv. *syringae* were selected for genome sequence analysis based on known differences in plant host range as demonstrated by pathogenicity assays in maize seedlings. It was reported (Gross and DeVay 1977) that strain HS191 (labeled as 5D430) caused water-soaked lesions in leaves within 48 h of inoculation to eventually result in the full range of holcus spot disease symptoms. In contrast, strain B301D did not cause disease symptoms in maize and showed insignificant multiplication in leaf tissue. This result for strain B301D is in sharp contrast to its ability to be highly virulent in immature cherry fruits (Scholz-Schroeder et al. 2001a) and to cause bacterial canker disease in cherry and apricot orchards (Gross et al. 1984).

Subsequently, it was shown that *N. benthamiana* was highly susceptible to a wide range of *P. syringae* strains, including B728a (Vinatzer et al. 2006). We therefore evaluated the virulence of strains B301D and HS191 relative to B728a in pathogenicity assays in *N. benthamiana* using both high (10^8^ CFU/mL) and moderate (10^6^ CFU/mL) inoculum concentrations. All three strains caused disease symptoms including formation of water-soaked lesions within 48 h of inoculation. Strain HS191 was comparable to B728a in virulence, whereas disease symptoms caused by strain B301D were less severe with lesions developing at a slower rate. Finally, we evaluated the ability of strains B301D and HS191 to cause disease symptoms in bean plants similar to B728a, a brown spot disease strain (Yu et al. 2013). Strain B301D and HS191 failed to cause disease symptoms in bean leaves. In contrast, strain B728a was pathogenic to bean and caused typical brown spot disease symptoms.

### General genomic features of *P. syringae* pv. *syringae* strains B301D and HS191

The genome of *P. syringae* pv. *syringae* B301D is composed of a circular chromosome 6,094,821 bp in size (Table1). In comparison, the chromosome of strain HS191 is slightly smaller at 5,950,211 bp, but HS191 also carries a 52,548 bp plasmid, pCG131 (Gonzalez et al. 1984). The G+C contents for the two genomes are 59.16% (B301D) and 58.96% (HS191) (Fig. S1), which is highly consistent with other *P. syringae* strains (Buell et al. 2003; Feil et al. 2005; Joardar et al. 2005). The B301D genome has a total of 5367 genes with 4375 genes with predicted functions and 125 RNA genes (Table1). Slightly fewer numbers of genes are found for the strain HS191 genome with a total of 5295 genes of which 4337 have predicted functions together with 122 RNA genes (Table1). Consequently, assigned functions for about 82% of the genes are observed for both *P. syringae* pv. *syringae* strains; the number of genes identified as encoding hypothetical proteins is limited to about 16% of the genes for both B301D and HS191. The total protein coding sequences are listed in Table1.

**Table 1:** General features of the *Pseudomonas syringae* pv. *syringae* B301D and HS191 genomes

Chromosome features^1^	Values	
	B301D	HS191
Genome size (bp)	6,094,819	5,950,211
Plasmid (bp)	0	1 (52,548)
DNA coding sequence (%)	88.59	88.82
GC content (%)	59.16	58.96
Number of total genes	5367	5295
Protein coding genes	5242	5173
rRNA genes (5S rRNA, 16S rRNA, 23S rRNA)	16 (6, 5, 5)	16 (6, 5, 5)
tRNA	65	66
Other RNA genes	44	40
Number of total RNA genes	125	122
Number of genes with assigned function	4375 (81.5%)	4337 (81.9%)
Number of genes without assigned function	867 (16.1%)	836 (15.8%)
Number of predicted enzymes	1285 (23.9%)	1257 (23.7%)

1 The features identified for each category were retrieved from IMG/ER after submission and annotation of genome data.

Plasmid pCG131 from strain HS191contains 61 annotated protein coding genes of which 25 encode hypothetical proteins and four genes encode transcriptional regulatory proteins.

### Phylogenetic analysis of sequenced strains B301D and HS191 with other *Pseudomonas* spp.

A phylogenetic tree evaluating the relationships of representative strains of *P. syringae* was constructed using Bayesian probability (Fig.1). The tree was assembled based on MLSA of 10 housekeeping genes (*acsA*, *aroE*, *dnaE*, *guaA*, *gyrB*, *mutL*, *ppsA*, *pyrC*, *recA*, and *rpoB*) that were previously shown to yield a robust comparison of relationships among species of *Pseudomonas* (Loper et al. 2012). The analysis included seven *P. syringae* strains with draft genome sequences that, despite being incomplete genomes, the 10 housekeeping gene sequences were available for use in the MLSA analysis. The phylogenetic tree shown in Figure1 was constructed by alignment of the 10 concatenated housekeeping genes by the maximum likelihood method. Within the phylogenetic tree, strains B301D and HS191 are clustered together with other genomospecies 1 strains of *P. syringae*. The genomospecies 3 strain (DC3000) and the genomospecies 2 strains (1448A, B076 and NCPPB 3335) are grouped in distinct clades within the tree consistent with earlier phylogenetic analysis of *P. syringae* (Gardan et al. 1999; Baltrus et al. 2014; Berge et al. 2014).

Based on the MLSA data, strain B301D is most closely related to strain B728a, which embodies the initial full genome sequence for genomospecies 1 (Feil et al. 2005). Strain HS191 is grouped with strain BRIP39023, a monocot strain that was originally isolated in Australia from wheat (Gardiner et al. 2013). Together with the genomes of strains SM and B64 from wheat (Dudnik and Dudler 2013a,b) and BRIP34876 and BRIP34881 from barley (Gardiner et al. 2013), the strains of *P. syringae* isolated from monocots form a subclade within the genomospecies 1 clade. Recently, the distinctiveness of *P. syringae* strains from grasses was supported by comparative genomic analysis of draft genomes that compared virulence-related genes (Dudnik and Dudler 2014b). A phylogenetic study by (Berge et al. 2014) using four housekeeping genes grouped B728a and related strains in *P. syringae* phylogroup two with five subgroups. We determined that strain B301D was grouped along with B728a in subgroup 2d and strain HS191 was grouped along with B64 in subgroup 2b (Berge et al. 2014).

### Comparison of the B301D and HS191 genomes to the B728a genome

**Figure 2:**
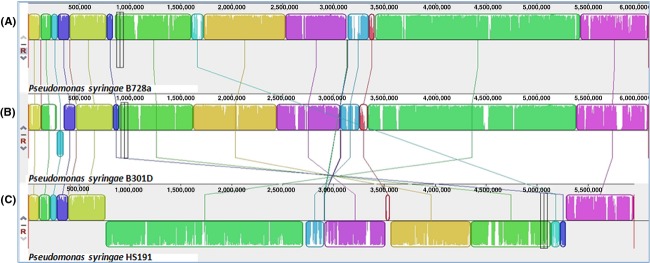
Pairwise alignment between the chromosomes of *Pseudomonas syringae* pv. *syringae* strains (A) B728a, (B) B301D, and (C) HS191 using MAUVE software (Darling et al. 2010). Colors depict conserved and highly related genomic regions and white areas identify unique or low identity regions. Blocks shifted below the center line indicate segments that align in the reverse orientation as inversions relative to reference strain B728a.

The chromosome organization of strains B301D and HS191 was compared to strain B728a by using the Progressive MAUVE algorithm. The B301D genome is largely collinear to the B728a genome (Fig.2). One prominent dissimilarity of the B301D genome to that of the other two strains is an inversion extending from locus tag PsyrB_01325 to 01650 (69,233 bp size), which is apparently due to the presence of a transposase at both these sites in the genome. A major deletion is observed in the B301D genome as compared to the genomes of strains B728a (Psyr_1426 to 1534; 109,171 bp size) and HS191 (PsyrH_22675 to 23150; 85,358 bp size). This region found lacking in B301D carries genes involved in type IV pilus formation as well as encoding hypothetical proteins.

Analysis of the chromosome organization for strain HS191 revealed several translocations and inversions relative to the genomes of strains B728a and B301D (Fig.2). A major rearrangement (inversion) is observed encompassing the middle portion of the HS191 chromosome extending from PsyrH_03465 to 23405 (4,518,882 bp size). This inversion is associated with the first (PsyrH_03435 to 03460) and fourth (PsyrH_23410 to 23440) copies of the rRNA operon in the HS191 chromosome, which like B301D and B728a carries a total of five copies of the rRNA operon. Chromosomal rearrangements commonly occur by recombination in bacteria with multiple copies of rRNA operons, and this phenomenon has been reported to frequently occur in host-specific *Salmonella* (Matthews et al. 2011). In addition, a secondary inversion in HS191 is observed from PsyrH_15245 to 15480 (34,694 bp size) as compared to the B728a and B301D chromosomes. This is a prophage 1 region present in the HS191 chromosome flanked by integrases. Genome arrangements in bacteria are useful as indicators of vertical inheritance (Darling et al. 2008), and the organization of the HS191 genome may prove to be indicative of distinct populations of pathovar *syringae*.

**Figure 3:**
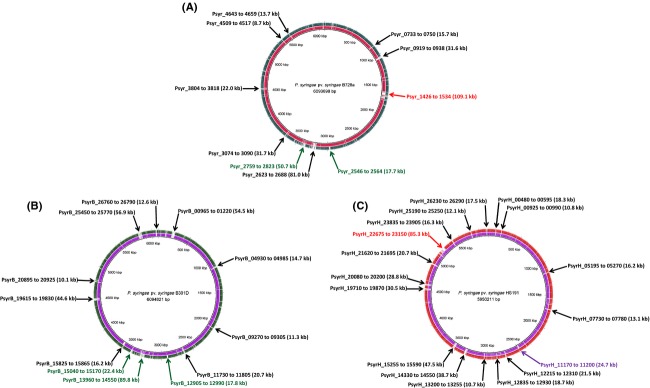
Circular representation of three genome comparisons using the BLAST Ring Image Generator (BRIG) software (Alikhan et al. 2011). The inner scales designate the coordinates in kilobase pairs (kbp). White spaces indicate regions with no identity to the reference genome. The gene clusters absent in B301D are indicated by a red arrow, clusters absent in HS191 are indicated by a green arrow, clusters absent in B728a are indicated by a purple arrow, and clusters absent in both the query genomes are indicated by a black arrow. (A) B728a genome (center) compared against the genomes of B301D (Ring 1 in red) and HS191 (Ring 2 in green). (B) B301D genome (center) compared against the genomes of B3728a (Ring 1 in purple) and HS191 (Ring 2 in green). (C) HS191 genome (center) compared against the genomes of B728a (Ring 1 in purple) and B301D (Ring 2 in red). Relative shading density (from darker to lighter) within each circle represents relative levels of nucleotide homology.

BRIG analysis was used to compare the genomes of B728a, B301D, and HS191 to identify large genome regions that are absent in one or more of these strains (Fig.3A–C). Seven B728a genomic regions, ranging in size from 8.7 to 81.0 kb, are absent in both strains B301D and HS191 (labeled in Fig.3A as Psyr_0733 to 0750, Psyr_0919 to 0938, Psyr_2623 to 2688, Psyr_3074 to 3090, Psyr_3804 to 3818, Psyr_4509 to 4517, and Psyr_4643 to 4659). Although these regions in B728a primarily carry genes of unknown function, three of the regions carry a type III effector gene (*avrRpm1*, Psyr_0738; *hopAF1*, Psyr_3813; *hopAB1*, Psyr_4659). It was noted, however, that the HS191 genome carries the *hopAF1* gene (PsyrH_07325), but lacks the surrounding genes observed in B728a (Psyr_3804 to 3818). In addition, the B301D genome lacks a 109.1 kb B728a region (Psyr_1426 to 1534) and an 85.3 kb HS191 region (PsyrH_22675 to 23150) containing several genes of unknown function, and a few genes associated with type IV secretion. A ∼50 kb region, associated with a temperate phage conserved in B728a (Psyr_2759 to 2823) and B301D (PsyrB_13920 to 14250), is absent in the HS191 genome. HS191 also lacks a second gene cluster of unknown function estimated to be ∼18 kb in strains B728a (Psyr_2546 to 2564) and B301D (PsyrB_12905 to 12990).

Similarly, when the genome of strain B301D was used as reference against the query genomes of strains B728a and HS191 (Fig.3B), nine regions are identified ranging from 10.1 to 56.9 kb in size that are not present in the query genomes (PsyrB_00965 to 01220, PsyrB_04930 to 04985, PsyrB_09270 to 09305, PsyrB_11710 to 11805, PsyrB_15825 to 15865, PsyrB_19615 to 19830, PsyrB_20895 to 20925, PsyrB_25450 to 25770, and PsyrB_26760 to 26790). Analysis of these B301D-specific regions revealed that one region (PsyrB_11730 to 11805) carries a series of type VI secretion genes, copper resistance genes that are duplicated in two regions (PsyrB_19770 to 19800, and PsyrB_25680 to 25710), and a gene cluster associated with a prophage (PsyrB_25460 to 25640). In addition, an 11.8 kb region of B728a (Psyr_1476 to 1488) is found within a 22 kb region of B301D (PsyrB_15040 to 15170), but is completely absent in HS191.

BRIG analysis with HS191 as the reference genome (Fig.3C) identified 15 gene clusters ranging in size from 10.7 to 47.5 kb (PsyrH_00480 to 00595, PsyrH_00925 to 00990, PsyrH_05195 to 05270, PsyrH_07730 to 07780, PsyrH_12215 to 12310, PsyrH_12835 to 12930, PsyrH_13200 to 13255, PsyrH_14330 to 14550, PsyrH_15255 to 15590, PsyrH_19710 to 19870, PsyrH_20080 to 20200, PsyrH_21620 to 21695, PsyrH_23835 to 23905, PsyrH_25190 to 25250, and PsyrH_26230 to 26290) that are absent in both the B728a and B301D genomes. Although most of these HS191 regions encode proteins of unknown function, some regions reveal a cluster of prophage genes (PsyrH_15245 to 15480), a type III effector gene (*hopW1*, PsyH_12235), and a region with two nonribosomal peptide synthetase (NRPS) genes (PsyrH_26255 and PsyrH_26265) of unknown function. A 25 kb region present in both HS191 (PsyrH_11170 to 11200) and B301D (PsyrB_15870 to 15885), but absent in B728a, contain genes associated with hemolysin-like activation and secretion.

**Figure 4:**
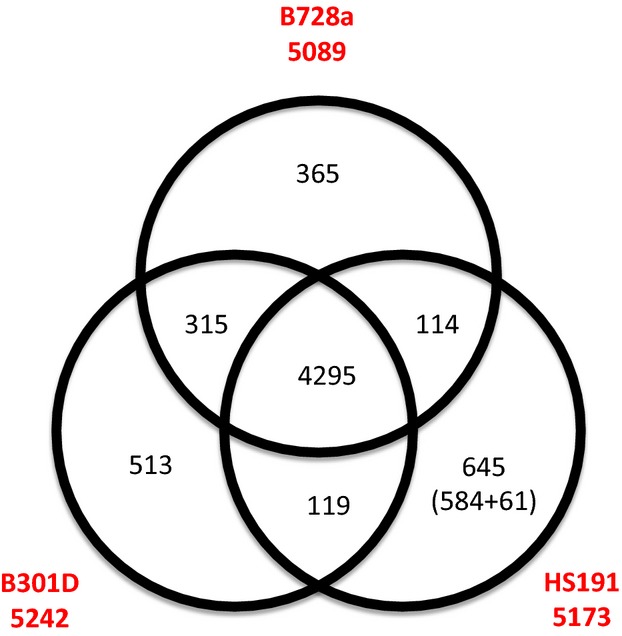
Venn diagram comparing the unique and shared genes between the genomes of B728a (top), B301D (left), and HS191 (right) using BLASTp. The numbers below the strain names identify the total number of protein coding genes within each genome. The strain HS191 genome has 645 unique genes, including 61 unique genes on plasmid pCG131. Genes that are conserved among all three strains are shown in the center of the diagram as the pangenome.

Horizontal gene transfer (HGT) plays a significant role in the diversification of lineages in *P. syringae* especially in regard to the evolution of defined pathovars (Nowell et al. 2014). BRIG analysis of the B728a, B301D, and HS191 genomes suggests that most of the unique gene regions (Fig.3) are associated with HGT as they frequently carried transposons, phage elements, and IS elements. The genome regions identified by BRIG analysis are largely composed of unique genes, ranging from 45% to 60% of the total number of unique genes in a given strain as depicted by a Venn diagram (Fig.4). In addition, BRIG analysis also was useful in identifying shared gene regions present in two strains, but absent in the other strain.

### Analysis of shared and unique genes in the B301D and HS191 genomes

A Venn diagram depicting the shared and unique genes of B301D and HS191 relative to B728a is presented in Figure4. The genomes of the three strains are composed of a large contingent of conserved shared genes estimated to be 4295 coding DNA sequences (CDS) that represent about 83% of each genome. Therefore, between 7% to 12% of the genes are unique among the genomes of the three strains based on a three-way best match using a reciprocal BLASTp search (Fig.4). The HS191 genome has more unique genes (645 genes) than either B301D (513 genes) or B728a (365 genes). Furthermore, the B728a and B301D genomes have a larger number of shared genes (315 genes) that are not shared with the HS191 genome. Strains B301D and HS191 share only 124 genes that are not present in B728a, whereas B728a and HS191 share 114 genes that are absent in B301D. It was observed, however, that a large proportion of the unique genes identified for both strains B301D (449 genes) and HS191 (566 genes) have gene homologs in other *Pseudomonas* species. The remaining unique genes from B301D (64 genes) and HS191 (79 genes) are mainly hypothetical protein and phage-related genes that did not match genes in other pseudomonad strains, but are found to have orthologs in other bacterial genera. Nearly half of the B301D genes categorized as unique (i.e., 244 genes) are of unknown function, whereas the other genes are mostly associated with phages (31 genes), transposases (31 genes), integrases (nine genes), transporters (16 genes), and regulators (15 genes). A prominent set of unique genes for B301D includes a 20.7 kb cluster of Type VI secretion genes (PsyrB_11730 to 11805) (Fig. S2). Likewise, the HS191 unique genes (excluding the genes from pCG131) are mostly categorized as unknown function (i.e., 273 genes), and other unique genes are associated with phages (47 genes), transposases (12 genes), integrases (14 genes), transporters (17 genes), and regulators (43 genes).

In the shared gene category for strains B301D and B728a, there are about 315 genes that mostly encode hypothetical proteins (111 genes), phage-related proteins (67 genes), transporters (22 genes) and transcriptional regulators (20 genes). One notable distinction of the B301D genome is the presence of two duplicated copies of a copper resistance gene cluster containing the *copABCDRS* genes (PsyrB_25680 to 25710; PsyrB_19770 to 19800) that are orthologs of a copper-resistance locus in the B728a (Psyr_1498 to 1493) genome (Feil et al. 2005) (Fig. S3). Consequently, we tested all three strains for copper resistance in vitro (Bender and Cooksey 1986). Despite a duplication of copper resistance genes in B301D there was no observable difference with B728a strain based on resistance to 0.8 mmol/L of copper sulfate, which corresponds to group II strains of *P. syringae* that tolerate copper sulfate (Bender and Cooksey 1986). In contrast, strain HS191 does not grow at a 0.8 mmol/L concentration of copper sulfate and this corresponds to a lack of the *copABCDRS* gene cluster. The two strains also harbor the *ahlI-ahlR* (PsyrB_07890 to 07895) (Fig. S4), which are a pair of genes required for production of an N-acyl-homoserine lactone that mediates quorum sensing in *P. syringae* (Quinones et al. 2004). The lack of *ahlI-ahlR* homologs in the monocot strain HS191 is consistent with observations that Poaceae-colonizing strains tend to lack a recognized quorum sensing system (Dudnik and Dudler 2014b). The *iaaM-iaaH* genes associated with the biosynthesis of indole-3-acetic acid (Feil et al. 2005) in B728a are observed in B301D (PsyrB_07455 to 07460) (Fig. S5), but are absent in the HS191 genome. It was also noted that B301D shares a urease gene cluster (PsyrB_10955 to 11005) with B728a (Psyr_2195 to 2205) that is associated with the conversion of urea to ammonia (Lan et al. 2006) (Fig. S6). In addition, a duplicate of this urease gene cluster is found in the genomes of all three strains (B728a, Psyr_4426 to 4436; B301D, PsyrB_22970 to 23020; and HS191, PsyrH_04065 to 04115). The HS191 and B728a genomes share a major gene cluster 85 kb in size (PsyrH_22675 to 23150) that carries 66 genes mostly of unknown function (45) and a Type IV secretion system including the *pilL-2* (PsyrH_22780) through *pilM-2* (PsyrH_22825) genes. There are 119 shared genes between the HS191 and B301D genomes of which most are designated as having unknown function.

A total of 15 sigma factors were previously identified in the B728a genome (Feil et al. 2005) and are conserved in the B301D genome. However, strain HS191 lacks the *ecf7* sigma factor gene, which encodes a FecI-type ECF sigma factor associated with iron stress and controls the ability of cells to swarm on semisolid surfaces (Thakur et al. 2013).

### Gene clusters conserved in all three stains that are associated with lifestyle

Because about 83% of the CDS in each of the genomes of the three *P. syringae* pv. *syringae* strains is composed of conserved genes, several gene clusters encoding features associated with pseudomonad lifestyle were cataloged. As shown for B728a (Feil et al. 2005), strains B301D and HS191 share gene clusters dedicated to the biosynthesis, secretion, and uptake of both the pyoverdine and achromobactin siderophores associated with ecological fitness due to their ability to facilitate extracellular iron uptake. The B728a ice nucleation gene (Psyr_1608, ∼4 kb in size) that is associated with the formation of an outer membrane protein that serves as a nucleus for ice crystal formation is conserved in B301D (PsyrB_07820) (Gross et al. 1984). In contrast, the ice nucleation gene is truncated in HS191 (PsyrH_18495), whereby it is missing nearly 2 kb of sequence in the center of the open reading frame (ORF) as compared to the gene in B728a and B301D. Accordingly, we tested the ice nucleation activity for all the three strains at −5°C (Lindow et al. 1978). Strains B728a and B301D show strong ice nucleation activity, whereas HS191 does not have ice nucleation activity at −5°C to show that the truncated ice nucleation gene (PsyrH_18495) is inactive.

Osmotolerance of *P. syringae* to limited water availability in the plant environment is promoted by the production of trehalose and *N*-acetylglutaminylglutamine amide (NAGGN), and by the accumulation of betaine (Li et al. 2013). The choline and betaine dehydrogenase enzymes, respectively encoded by the *betA* and *betB* genes, synthesize betaine in pseudomonads upon uptake of exogenous choline (Kurz et al. 2010). Both the *betA* and *betB* genes are conserved in strains B301D (PsyrB_24500 to 24505) and HS191 (PsyrH_24260 to 24265), as are the genes attributed to the synthesis of trehalose and NAGGN in strain B728a (Feil et al. 2005; Kurz et al. 2010).

Extracellular polysaccharide (EPS) production is associated with virulence in *P. syringae* pv. *syringae* due to their role in adhesion to plant surfaces and biofilm formation (O’Brien et al. 2011b). Alginate, levan, and Psl are three distinct EPS’s produced by B728a (Yu et al. 2014), and the biosynthesis and secretion genes associated with their production are conserved in the genomes of B301D and HS191. Finally, strains B301D and HS191 contain a complete gene cluster for Type VI secretion of the HSI type (Sarris et al. 2010) that is syntenic with the 29.9-kb cluster of genes observed in strain B728a (Records 2011). However, a second complete Type VI gene cluster (PsyrB_11730 to 11805) is present in B301D that is homologous to the HSI-1 Type VI cluster of *P. syringae* pv. *tomato* DC3000 (Sarris et al. 2010).

### Type III secretion effectors

The Type III secretion system is encoded by a series of *hrp/hrc* genes within a 26 kb pathogenicity island in B728a and is well-known for secreting effector proteins that modulate virulence (O’Brien et al. 2011b). Relative to the B728a genome, the B301D *hrp/hrc* gene region is strictly conserved as a syntenic block whereas the HS191 genome is missing about a 3 kb region associated with two genes of unknown function (i.e., Psyr_1203 and Psyr_1204).

A major focus of genomic studies of *P. syringae* strains has been the relationship of type III effector content and its impact on host range and evolutionary processes critical to plant pathogenesis (Lindeberg et al. 2008; O’Brien et al. 2011b). Accordingly, the type III effector content of the B301D and HS191 genomes was identified by searches to a comprehensive list of known *P. syringae* effectors (Baltrus et al. 2011; Bart et al. 2012) and compared to the effector profile of B728a (Vinatzer et al. 2006). In addition to the 22 B728a effectors previously identified (Vinatzer et al. 2006), two additional (i.e., *hopAN1* and *hopAP1*) effector genes were found (Table2). The B301D genome with a total of 19 effector genes has the fewest effectors, whereas the HS191 genome carries a total of 25 effector genes. It also was observed that 15 B728a effector genes occur in both the B301D and HS191 genomes. Of these effectors, *hopAA1*, *avrE1*, *hopM1*, *hopI1*, and *hopAH1* are present in all three strains and are consistent with the observation (Baltrus et al. 2011) that these five effector gene families routinely are found in *P. syringae* regardless of genomospecies boundaries. Furthermore, three of these genes (i.e., *hopAA1*, *avrE1*, and *hopM1*) are located within the conserved effector locus (CEL) flanking the Type III pathogenicity island in disparate *P. syringae* strains (Alfano et al. 2000; O’Brien et al. 2011a), and it has been demonstrated that deletion of the CEL region leads to a pronounced loss in virulence (Alfano et al. 2000; Kvitko et al. 2009). The exchangeable effector locus (EEL) that also flanks the Hrp Type III gene cluster of B728a carriers three effector genes (i.e., *avrB3*, *hopX1*, and *hopZ3*) that are absent in both strains B301D and HS191. Instead, the EEL regions for these two strains carry a single effector gene called *hopA2*, which corresponds to the EEL class IB as described previously for strain B301D (Charity et al. 2003). The *hopA2* effector gene also occurs in the EEL region of *P. syringae* pv. *averrhoi* HL1 and was reported to contribute to virulence in susceptible carambola plants (Lin et al. 2006).

**Table 2:** Effector gene contents of *Pseudomonas syringae* pv. *syringae* strains B728a, B301D and HS191

SI. no.	Organisms	Effectors genes	B728a locus_tag	B301D locus_tag	HS191 locus_tag
1	*P. syringae* pv. *syringae* B728a	*avrB3*	Psyr_1219	–	–
2		*avrE1*	Psyr_1188	PsyrB_06260	PsyrH_20365
3		*avrPto1*	Psyr_4919	–	–
4		*avrRpm1*	Psyr_0738	–	–
5		*hopAA1*	Psyr_1183	PsyrB_06235	PsyrH_20390
6		*hopAB1*	Psyr_4659	–	–
7		*hopAC1*	Psyr_0527	PsyrB_02970	PsyrH_02665
8		*hopAE1*	Psyr_4269	PsyrB_22195	PsyrH_04865
9		*hopAF1*	Psyr_3813	–	PsyrH_07325
10		*hopAG1*	Psyr_0778	PsyrB_04160	PsyrH_22420
11		*hopAH1*	Psyr_0779	PsyrB_04165	PsyrH_22410
12		*hopAH2*	Psyr_3123	PsyrB_16095	PsyrH_10955
13		*hopAI1*	Psyr_0785	PsyrB_04195	PsyrH_22380
14		*hopAJ2*	Psyr_4357	PsyrB_22625	PsyrH_04450
15		*hopAK1*	Psyr_3839	PsyrB_19950	PsyrH_07200
16		*hopAN1*	Psyr_0465	PsyrB_02660	PsyrH_02345
17		*hopAP1*	Psyr_1890	PsyrB_09325	–
18		*hopH1*	Psyr_1889	PsyrB_09320	PsyrH_20075
19		*hopI1*	Psyr_4326	PsyrB_22470	PsyrH_04610
20		*hopJ1*	Psyr_1017	PsyrB_05375	PsyrH_21240
21		*hopL1*	Psyr_2631	–	–
22		*hopM1*	Psyr_1186	PsyrB_06250	PsyrH_20375
23		*hopX1*	Psyr_1220	–	–
24		*hopZ3*	Psyr_1224	–	–
25	*P. syringae* pv. *syringae* B301D	*hopA2*	–	PsyrB_06425	PsyrH_20205
26	*P. syringae* pv. *maculicola* ES4326	*hopAL1*	–	PsyrB_08740	–
27	*P. syringae* Cit 7	*hopAX1*	–	–	PsyrH_18845
28	*P. syringae pv. syringae* B64	*hopAZ1*	–	–	PsyrH_17410
29	*P. syringae pv. syringae* B64	*hopBA1*	–	–	PsyrH_12900
30	*P. syringae pv. aptata* DSM 50252	*hopBC1*	–	PsyrB_08275	PsyrH_18105
31	*P. syringae* pv. *tomato* DC3000	*hopC1*	–	–	PsyrH_20070
32	*P. syringae* pv. *tomato* T1	*hopR1*	–	–	PsyrH_12260
33	*P. syringae* pv. *maculicola* ES4326	*hopW1*	–	–	PsyrH_12235
34	*P. syringae pv. syringae* B64	*hopZ1*	–	–	PsyrH_20105
Total effectors			24/34	19/34	25/34

– indicates absence.

The *hopAL1* effector gene is present in B301D and not in either B728a or HS191. HopAL1 is reported to function as a phosphothreonine lyase that contributes to virulence of *P. syringae* pv. *tomato* DC3000 by inactivating mitogen-activated protein kinases (Beckers et al. 2009). Another distinguishing characteristic for B301D is that it lacks *hopAF1* that is commonly found in *P. syringae* genomes. Seven effector genes (i.e., *hopAX1*, *hopAZ1*, *hopBA1*, *hopC1*, *hopR1*, *hopW1*, and *hopZ1*) are present in the genome of HS191 and not the other two strains. The *hopH1* and *hopC1* genes in *P. syringae* pv. *tomato* DC3000 are linked with a possible pyocin gene insert speculated to be associated with pyocin mobilization (Lindeberg et al. 2008); these two effector genes in HS191 are linked with phage-associated genes. The *hopW1* effector gene is associated with a plasmid in *P. syringae* pv. *maculicola* ES4326 (Stavrinides and Guttman 2004), but in HS191 *hopW1* is located on the chromosome and is likewise linked to the resolvase-integrase-*exeA* trio of genes. The HopW1 effector was recently demonstrated to disrupt the actin cytoskeleton of host plants to promote a strong virulence response (Kang et al. 2014). The *hopZ1* gene is found in several pathovars of *P. syringae* with the HopZ1 effector carrying a cysteine-protease catalytic core (Ma et al. 2006). Thus, the effector composition of strain HS191 is complex especially in regard to their functions in controlling host range.

### Comparative analysis of genes associated with toxin biosynthesis

The production of two classes of cyclic lipopeptide phytotoxins, called syringomycins and syringopeptins, is characteristic of genomospecies 1 strains of *P. syringae* (Scholz-Schroeder et al. 2003; Young 2010). The toxins are synthesized by a classical NRPS mechanism, which is encoded by two large genomic regions located adjacent to one another and together encompass a 145-kb expanse of the chromosome (Scholz-Schroeder et al. 2001b). The *syr* gene clusters dedicated to syringomycin production in both strains B301D and HS191 are both about 55 kb in size and collinear with the organization of the *syr* gene cluster of strain B728a (Feil et al. 2005). This conserved *syr* gene organization corresponds to earlier reports that these strains produced structurally identical lipodepsinonapeptide forms of syringomycin (Segre et al. 1989; Grgurina et al. 2002).

**Figure 5:**
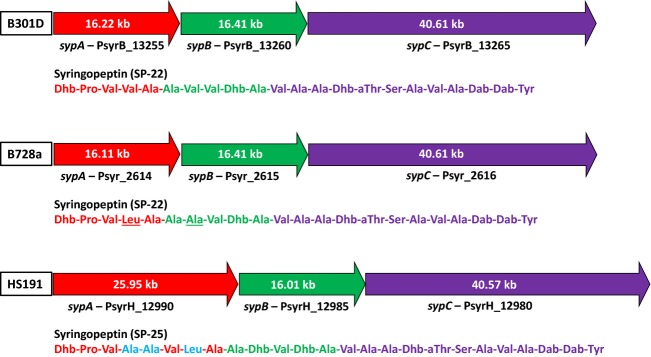
Comparison of the organization of the syringopeptin nonribosomal peptide synthetase genes *sypA*, *sypB*, *sypC* in strains B301D (top), B728a (center), and HS191 (bottom). Strains B301D and B728a produce SP22 (Grgurina et al. 2002) and HS191 produces SP25 (Ballio et al. 1991). The amino composition for SP22 and SP25 are shown below the respective synthetase genes; the color of the amino acid corresponds to the specific *syp* synthetase gene. The *sypA* gene of HS191 encodes NRPS modules for three additional amino acids in SP25 as shown in blue; B728a compared to B301D is modified in amino acids at positions four and seven (underlined). The size of the bar approximately corresponds to gene size.

The syringopeptin class of toxins is encoded by one of the largest NRPS systems known to occur in bacteria (Scholz-Schroeder et al. 2003). A distinctive feature of the B301D *syp* gene cluster, which encompassed about an 80 kb DNA region, is the presence of a trio of NRPS synthetase genes called *sypA*, *sypB*, and *sypC* that together encode 22 amino acid-activating modules (Fig.5). These activation modules account for the incorporation of all 22 amino acids found in the syringopeptin structure called SP22; strain B728a produces a modified form called SP_22_Phv with Leu and Ala replacing the two Val residues in SP22 at positions four and seven (Grgurina et al. 2002). Strain HS191 was reported earlier (Ballio et al. 1991) to produce a larger form of syringopeptin called SP25, which contains three additional structural amino acids in the peptide chain. Sequence analysis of the three *syp* synthetase genes of HS191 resolved the organization of the 25 activation modules dedicated to SP25 production. The *sypA* synthetase gene of HS191 at 26 kb in size encodes all three additional amino acid activation modules associated with synthesis of SP25; altogether 25 modules are accounted for based on the structural organization of the three HS191 Syp synthetases (Fig.5). The modules unique to SP25 are located at positions four (Ala), five (Ala), and seven (Leu) on the *SypA* synthetase based on analysis of predicted amino acid substrate specificity (Röttig et al. 2011). The *syp*C gene of HS191, which encodes 12 activation modules, is conserved in all three *P. syringae* strains. Overall, the occurrence and positional organization of the syringopeptin gene cluster is conserved among strains B728a, B301D, and HS191 despite observed differences in the *sypA* synthetase gene. In comparing the B301D to the draft genome of B301D-R, we observed that the *sypC* gene of B301D-R (PssB301D_02641) was missing a large 22.2 kb section as compared to B301D (PsyrB_13265); this illustrates deficiencies in quality and completeness of the B301D-R draft genome (Dudnik and Dudler 2014a).

**Figure 6:**
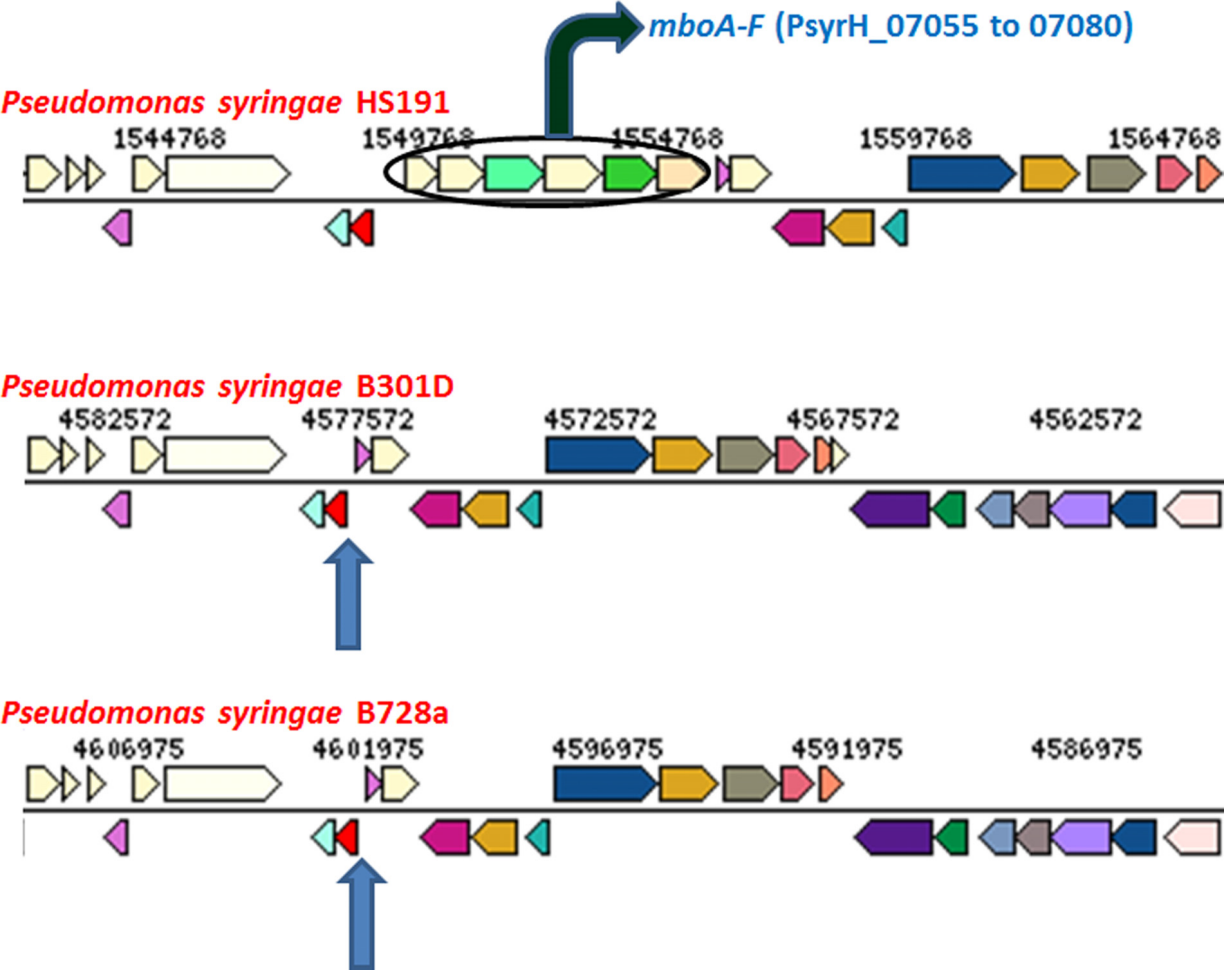
Comparison of the region in strain HS191 carrying the *mbo* operon (*mboA* to *mboF*) responsible for mangotoxin production with the corresponding regions in strains B728a and B301D (blue arrows indicate predicted location) that do not produce mangotoxin and lack the *mbo* operon. Colors indicate ortholog groups and the size of the bar approximately corresponds to gene size. This comparison was done using JGI-IMG/ER (https://img.jgi.doe.gov/cgi-bin/w/main.cgi).

Mangotoxin is an antimetabolite toxin that is distributed within specific genomospecies 1 populations of *P. syringae* and was first associated with strains from diseased mango trees (Gutiérrez-Barranquero et al. 2013). Mangotoxin is a potent inhibitor of ornithine *N*-acetyl-transferase in the arginine biosynthesis pathway and thereby contributes significantly to virulence in host plants (Carrión et al. 2012, 2013). The mangotoxin biosynthesis apparatus is composed of a cluster of six *mbo* genes arranged in a 5.8 kb operon. It was reported that strain B728a is not a producer of mangotoxin and did not harbor the *mbo* operon (Carrión et al. 2012). Likewise, the B301D genome does not carry the *mbo* operon and thereby cannot produce mangotoxin (Fig.6). The HS191 genome, however, carries the full complement of mangotoxin biosynthesis genes. The draft sequences of the five monocot strains shown in Figure1 that are phylogenetically related to HS191 all carry the intact *mbo* operon. Accordingly, it is postulated that the monocot strains of pathovar *syringae* represent a distinct phylogroup that is distinguished by the ability to produce both syringopeptin SP25 and mangotoxin.

Syringolin is a fourth phytotoxin associated specifically with genomospecies 1 strains of *P. syringae*. The toxin is the product of a mixed NRPS and polyketide biosynthetic process that contributes to virulence by causing irreversible proteasome inhibition (Schellenberg et al. 2010). Comparison of the nearly 25-kb *syl* gene cluster for syringolin production showed that it is conserved in the genomes of strains B301D, HS191, and B728a (Amrein et al. 2004).

In addition to NRPS gene systems associated with phytotoxin production, strains B301D and HS191 both harbor the full complement of the syringofactin *syf* genes required for biosurfactant production in B728a (Hockett et al. 2013), and a complete set of pyoverdine *pvd* genes required for siderophore production (Feil et al. 2005). Both strains also carry two clusters of genes previously identified in the B728a genome (Feil et al. 2005) encoding NRPS components of unknown function (PsyrB_08785 to 08805 in B301D, corresponding to PsyrH_17575 to 17585 in HS191, and PsyrB_19205 in B301D, corresponding to PsyrH_07785 in HS191). The NRPS module encoded by Psyr_4662 in B728a is identified as PsyrH_23195 in HS191, but this NRPS gene of unknown function is not found in the genome of B301D. Finally, the *mgo* operon, including the *mgoA* NRPS gene (PsyrB_26220 in B301D, and PsyrH_25690 in HS191), is conserved in strains B301D and HS191. In *Pseudomonas entomophila*, the *mgo* operon was called *pvfABCD* and found to be conserved in many *Pseudomonas* species (Vallet-Gely et al. 2010). Recently, it was proposed that a metabolite produced by the *mgo* operon regulated biosynthesis of mangotoxin in strains that carry the *mbo* gene cluster (Carrión et al. 2014).

### Mobile genetic elements

The genomes of strains B728a, B301D and HS191 were analyzed for IS elements using the IS Finder (http://www-is.biotoul.fr/) database and Blast searches using a stringent *E* value threshold (below e^−100^) as described earlier (Wu et al. 2011). The complete list of putative IS elements identified are observed in Table S1 and encompass 10 distinct IS groups. Searches at a higher threshold E score yielded partial or remnants of IS elements in these three genomes. The presence of IS elements is linked to mobilization events and genomic rearrangements in pseudomonads (Lindeberg et al. 2008). The most IS elements are observed in the B728a genome which contains 32 complete IS elements spanning five families (i.e., IS3, IS5, IS66, IS630, and IS1182) to include seven groups. The number of IS elements for strain B728a is considerably larger than several *Pseudomonas fluorescens* genomes that were reported to carry up to 20 transposons (Loper et al. 2012). In comparison, strain B301D has 16 complete IS elements in four families (i.e., IS3*,* IS66*,* IS630*,* and IS1182), and HS191 has only six complete IS elements (two in the IS3 and four in the IS5 families). The majority of the IS elements in B728a are present in multicopies, especially members of the IS3 family (i.e., ISpsy8, ISpsy9, and ISpsy28). Of the IS groups, only ISpsy9 is present in all three genomes of *P. syringae*. The IS elements in five groups (i.e., ISpsy8, ISpsy9, ISpsy28, ISPpu19, and ISpsy27) are found in both the B728a and B301D genomes. The IS2000 and ISps1 groups are exclusive to the genome of the monocot strain HS191.

Using the Prophage Finder tool, the genomes of both strains B301D and HS191 are predicted to encode prophage and tailocin structures (Table S2). In addition, the program identified short regions in their genomes that contain remnants of phage gene clusters that are lineage specific. Two complete prophage gene clusters in strain B301D were identified. The largest B301D prophage gene cluster is estimated to be 47.3 kb in size and contain 67 genes (PsyrB_13920 to 14250) that closely resembles an orthologous prophage gene cluster in B728a (Yu et al. 2013) and is inserted in the same region of both strains. The second B301D prophage gene cluster is estimated to be 28.7 kb in size with 37 genes (PsyrB_25460 to 25640) and, although it is not present in the genomes of strains B728a and HS191, the gene cluster is orthologous to the prophage six cluster identified in *P. syringae* pv. *phaseolicola* 1448A (Joardar et al. 2005). The strain HS191 genome carries a 34.2 kb prophage gene cluster (PsyrH_15245 to 15480) with 47 genes and is most closely related to a prophage genes of *Pseudomonas savastanoi* pv. *savastanoi* strain NCPPB 3335 (Rodriguez-Palenzuela et al. 2010) (Table S2). All of these prophage gene clusters in B301D and HS191 contained genes for DNA packaging and head morphogenesis, tail synthesis, immunity, phage replication, and lysis, which are indicative of genesis of complete phage particle structures.

Tailocin gene clusters of about 29 kb are found in the genomes of B301D (PsyrB_23745 to 23915; 35 genes) and HS191 (PsyrH_23505 to 23680; 36 genes) (Table S2) that closely resembled the tailocin gene cluster previously identified in B728a (Yu et al. 2013). All of these tailocin clusters lack genes for head formation, but closely resemble the R-type syringacin class of bacteriocins that were previously described in *P. syringae* (Hockett et al. 2014).

### Analysis of the HS191 plasmid pCG131

**Figure 7:**
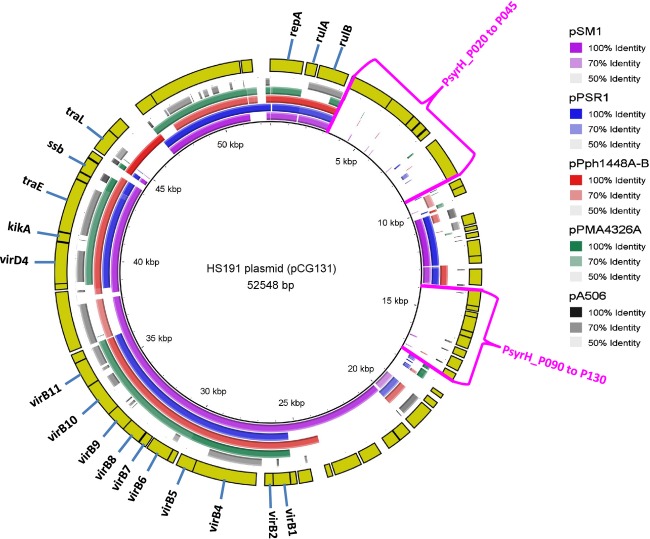
Circular representation of plasmids from pathovars of *Pseudomonas syringae* against HS191 plasmid pCG131 using the BLAST Ring Image Generator (BRIG) software (Alikhan et al. 2011). The inner scales designate the coordinates in kilobase pairs (kbp). Ring 1 = *P. syringae* pv. *syringae* plasmid pSM1 (purple); Ring 2 = *P. syringae* pv. *syringae* plasmid pPSR1 (blue); Ring 3 = *P. syringae* pv. *phaseolicola* pPph1448A-B (red); Ring 4 = *P. syringae* pv. *maculicola* pPMA4326A (green); Ring 5 = *Pseudomonas fluorescens* pA506 (black); Ring 6 = coding DNA sequence (CDS) of HS191 plasmid (yellow). The areas in pCG131 not occurring in the four plasmids used for comparison are indicated by red brackets. Relative shading density (from darker to lighter) within each circle represents relative levels of nucleotide homology.

The HS191 plasmid pCG131 was mapped using BRIG analysis to the *P. syringae* plasmids pSM1 (Dudnik and Dudler 2013b), pPSR1 (Sundin et al. 2004), pPph1448A-B (Joardar et al. 2005), pPMA4326A (Stavrinides and Guttman 2004), and *P. fluorescens* pA506 (Stockwell et al. 2013) that share large conserved DNA regions (Fig.7). The pCG131 plasmid is most similar to pSM1 followed by pPph1448A-B, but it is evident pCG131 represents a genetic mosaic composed of DNA regions seen in plasmids from diverse strains of *P. syringae* (Ma et al. 2007). The pCG131 plasmid has a *repA* gene (PsyrH_P005), which is distinctive for members of the *P. syringae* pPT23A-like family of replicons and is essential for plasmid replication (Stavrinides and Guttman 2004). The transmissibility of pCG131 (Gonzalez et al. 1984) is supported by the presence of a mobilization-like gene (PsyrH_P300) and conjugal transfer gene *traE* (PsyrH_P265). A *kikA* gene encoding a killer protein for plasmid maintenance (PsyrH_P260) also is present (Holcik and Iyer 1997). A prominent pCG131 region that is conserved in all four reference plasmids is associated with a type IV secretion system encoded by homologs to *virB1* (PsyrH_P185) through *virB11* (PsyrH_P235) involved in pilus formation and by *virD4* (PsyrH_P255). Accordingly, an earlier study using a macroarray to evaluate hybridization to a series of genes from pPT23A-type plasmids categorized pCG131 to have a complete type IVA secretion system (Zhao et al. 2005).

The biological significance of HS191 harboring the pCG131 plasmid appears to be based on the presence of genes that promote fitness of the bacterium in the plant environment. For example, pPT23A-type plasmids frequently contain UV resistance genes linked closely with integrase, resolvase and chemotaxis genes (Zhao et al. 2005) that appear to promote epiphytic fitness. Accordingly, immediately downstream of the *repA* gene (Fig.7), pCG131 carries the *rulA* (PsyrH_P010) and *rulB* (PsyrH_P015) genes that encode DNA polymerases for repair of UV damage (Sundin and Murillo 1999). Closely associated are integrase (PsyrH_P090) and chemotaxis (PsyrH_P045) genes to form the core of a possible fitness island. Associated with this region are two distinct areas of pCG1331 that are absent in all five reference plasmids with the first area extending from PsyrH_P020 to P045 and the second from PsyrH_P090 to P130 (Fig.7). The functions of these two plasmid areas are unknown as they are composed largely of genes encoding hypothetical proteins. Although it was previously reported (Zhao et al. 2005) that pCG131 carried homologs of two Type III effector genes that are now called *hopAM1–2* and *hopZ1*, no *hop* effector genes were identified on the pCG131plasmid. Furthermore, just the *hopZ1* gene was found to occur on the HS191 chromosome (Table2). In addition, PsyrH_P050 and PsyrH_P055 encode modules for the antitoxin RelB and RelF, respectively, that likely promote plasmid maintenance in bacterial populations (Wang and Wood 2011). Finally, an HCP1 family type VI secretion system effector (PsyrH_P150) (Zhou et al. 2012), a LuxR family transcriptional regulator (PsyrH_P160), and an ABC transporter gene (PsyrH_P165) are located in a pCG131 gene cluster as is also observed for pSM1.

## Conclusions

Complete genomes of *P. syringae* pv. *syringae* strains B301D and HS191 were generated by 454 pyrosequencing, Illumina, and OpGen technology for mapping KpnI sites. Intrapathovar comparisons of the genomes of B728a to the complete genomes of *P. syringae* pv. *syringae* strains B301D and HS191, originating, respectively, from dicot and monocot plant hosts, helped delineate traits shared by pathovar *syringae* strains that are critical to adaptation and survival in the plant environment. Accordingly, the shared genes for the three pathovar *syringae* strains encompassed about 83% of each genome, and included genes dedicated to siderophore biosynthesis, osmotolerance, and EPS production essential to biofilm formation. Whole genome analysis also revealed accessory genes encoding traits distinctive for individual strains differing in host specificity. Variations in the composition of type III effector content are observed for the three strains analyzed that likely influenced host range. The HS191 genome, with a total of 25 effector genes, has the largest contingent of effector genes of which seven are specific to this monocot strain. Toxin production is another major trait associated with virulence of *P. syringae* pv. *syringae*, and HS191 is distinguished by the production of syringopeptin SP25 and mangotoxin. In summary, resolution of the complete genome of two additional strains of pathovar *syringae* provides a foundation to further explore diversification of lineages among strains classified as genomospecies 1 and associated with diverse environmental reservoirs.

## Supporting Information

**Figure S1.** Circular representation using CGView of the *Pseudomonas syringae* B301D chromosome, and the HS191 chromosome plus plasmid pCG131. Circles from center to outside: first, GC skew; second, GC content; and third, predicted coding DNA sequences (CDS) of chromosome on leading and lagging strands, respectively.

**Figure S2.** Type VI gene cluster (HSI-1) present in B301D (PsyrB_11730 to 11805) were checked to match a particular ortholog neighborhood, and this gene cluster were missing in both B728a and HS191 (position shown by blue arrow). Colors indicate ortholog groups and the size of the bar approximately corresponds to gene size. This comparison was done using JGI-IMG/ER (https://img.jgi.doe.gov/cgi-bin/w/main.cgi).

**Figure S3.** Gene ortholog neighborhood of copper resistance gene cluster, *copABCDRS*, of B728a (Psyr_1498 to 1493) that is duplicated in B301D (PsyrB_19770 to 19800; PsyrB_25680 to 25710); this gene cluster is absent in HS191. Colors indicate ortholog groups and the size of the bar approximately corresponds to gene size. This comparison was done using JGI-IMG/ER (https://img.jgi.doe.gov/cgi-bin/w/main.cgi).

**Figure S4.** The *ahlI-ahlR* pair of genes responsible for quorum sensing that are conserved in B728a (Psyr_1621 to 1622) and B301D (PsyrB_07890 to 07895), but in absent in HS191 (position shown by blue arrow). Colors indicate ortholog groups and the size of the bar approximately corresponds to gene size. This comparison was done using JGI-IMG/ER (https://img.jgi.doe.gov/cgi-bin/w/main.cgi).

**Figure S5.** Two genes required for indole acetic acid (IAA) production, *iaaH* and *iaaM*, are present in B728a (Psyr_1536 to 1537) and B301D (PsyrB_07455 to 07460), but absent in HS191 (position shown by blue arrow). Colors indicate ortholog groups and the size of the bar approximately corresponds to gene size. This comparison was done using JGI-IMG/ER (https://img.jgi.doe.gov/cgi-bin/w/main.cgi).

**Figure S6.** Urease gene cluster responsible for conversion of urea to ammonia, present in B728a (Psyr_2195 to 2205) and B301D (PsyrB_10955 to 11005) were checked to match a particular ortholog neighborhood, and this gene cluster were missing in HS191 (position shown by blue arrow). Colors indicate ortholog groups and the size of the bar approximately corresponds to gene size. This comparison was done using JGI-IMG/ER (https://img.jgi.doe.gov/cgi-bin/w/main.cgi).

**Figure S7.** De novo assembly of next-generation sequencing data generates scaffolds (12 scaffolds of B301D [A] and six scaffolds of HS191 [B]) were converted into restriction maps by in silico restriction enzyme digestion. Subsequently, the sequencing-derived scaffolds are matched to the distance between restriction enzyme sites in the optical fragments in the KpnI restriction maps. Finally, the scaffolds were extended and linked based on matches to the KpnI optical map to form complete genomes maps for strains B301D (A), and HS191 plus plasmid pCG131 (B).

**Table S1.** IS element comparison for the *Pseudomonas syringae* pv. *syringae* strains B728a, B301D and HS191. **Table S2.** Prophage finder of *Pseudomonas syringae* pv. *syringae* strains B728a, B301D and HS191.

## References

[b1] Alfano JR, Charkowski AO, Deng WL, Badel JL, Petnicki-Ocwieja T, van Dijk K (2000). The *Pseudomonas syringae* Hrp pathogenicity island has a tripartite mosaic structure composed of a cluster of type III secretion genes bounded by exchangeable effector and conserved effector loci that contribute to parasitic fitness and pathogenicity in plants. Proc. Natl. Acad. Sci. USA.

[b2] Alikhan NF, Petty NK, Ben Zakour NL, Beatson SA (2011). BLAST Ring Image Generator (BRIG): simple prokaryote genome comparisons. BMC Genom.

[b3] Amrein H, Makart S, Granado J, Shakya R, Schneider-Pokorny J, Dudler R (2004). Functional analysis of genes involved in the synthesis of syringolin A by *Pseudomonas syringae* pv. *syringae* B301 D-R. Mol. Plant Microbe Interact.

[b4] Ballio A, Barra D, Bossa F, Collina A, Grgurina I, Marino G (1991). Syringopeptins, new phytotoxic lipodepsipeptides of *Pseudomonas syringae* pv. *syringae*. FEBS Lett.

[b5] Baltrus DA, Nishimura MT, Romanchuk A, Chang JH, Mukhtar MS, Cherkis K (2011). Dynamic evolution of pathogenicity revealed by sequencing and comparative genomics of 19 *Pseudomonas syringae* isolates. PLoS Pathog.

[b6] Baltrus D, Hendry T, Gross DC, Lichens-Park A, Kole C, Hockett K (2014). Ecological genomics of *Pseudomonas syringae*. Genomics of plant-associated bacteria.

[b7] Bart R, Cohn M, Kassen A, McCallum EJ, Shybut M, Petriello A (2012). High-throughput genomic sequencing of cassava bacterial blight strains identifies conserved effectors to target for durable resistance. Proc. Natl. Acad. Sci. USA.

[b8] Beckers GJM, Jaskiewicz M, Liu YD, Underwood WR, He SY, Zhang SQ (2009). Mitogen-activated protein kinases 3 and 6 are required for full priming of stress responses in *Arabidopsis thaliana*. Plant Cell.

[b9] Bender CL, Cooksey DA (1986). Indigenous plasmids in *Pseudomonas syringae* pv. *tomato*: conjugative transfer and role in copper resistance. J. Bacteriol.

[b10] Bender CL, Alarcon-Chaidez F, Gross DC (1999). *Pseudomonas syringae* phytotoxins: mode of action, regulation, and biosynthesis by peptide and polyketide synthetases. Microbiol. Mol. Biol. Rev.

[b11] Berge O, Monteil CL, Bartoli C, Chandeysson C, Guilbaud C, Sands DC (2014). A user’s guide to a data base of the diversity of *Pseudomonas syringae* and its application to classifying strains in this phylogenetic complex. PLoS One.

[b12] Buell CR, Joardar V, Lindeberg M, Selengut J, Paulsen IT, Gwinn ML (2003). The complete genome sequence of the *Arabidopsis* and tomato pathogen *Pseudomonas syringae* pv. *tomato* DC3000. Proc. Natl. Acad. Sci. USA.

[b13] Bull CT, Clarke CR, Cai R, Vinatzer BA, Jardini TM, Koike ST (2011). Multilocus sequence typing of *Pseudomonas syringae* sensu lato confirms previously described genomospecies and permits rapid identification of *P. syringae* pv. *coriandricola* and *P. syringae* pv. *apii* causing bacterial leaf spot on parsley. Phytopathology.

[b14] Cai R, Yan S, Liu H, Leman S, Vinatzer BA (2011). Reconstructing host range evolution of bacterial plant pathogens using *Pseudomonas syringae* pv. *tomato* and its close relatives as a model. Infect. Genet. Evol.

[b15] Carrión VJ, Arrebola E, Cazorla FM, Murillo J, de Vicente A (2012). The *mbo* operon is specific and essential for biosynthesis of mangotoxin in *Pseudomonas syringae*. PLoS One.

[b16] Carrión VJ, Gutiérrez-Barranquero JA, Arrebola E, Bardaji L, Codina JC, de Vicente A (2013). The mangotoxin biosynthetic operon (*mbo*) is specifically distributed within *Pseudomonas syringae* genomospecies 1 and was acquired only once during evolution. Appl. Environ. Microbiol.

[b17] Carrión VJ, van der Voort M, Arrebola E, Gutiérrez-Barranquero JA, de Vicente A, Raaijmakers JM (2014). Mangotoxin production of *Pseudomonas syringae* pv. *syringae* is regulated by MgoA. BMC Microbiol.

[b18] Charity JC, Pak K, Delwiche CF, Hutcheson SW (2003). Novel exchangeable effector loci associated with the *Pseudomonas syringae hrp* pathogenicity island: evidence for integron-like assembly from transposed gene cassettes. Mol. Plant Microbe Interact.

[b19] Darling AE, Miklos I, Ragan MA (2008). Dynamics of genome rearrangement in bacterial populations. PLoS Genet.

[b20] Darling AE, Mau B, Perna NT (2010). progressiveMauve: multiple genome alignment with gene gain, loss and rearrangement. PLoS One.

[b21] Dudnik A, Dudler R (2013a). Non contiguous-finished genome sequence of *Pseudomonas syringae* pathovar *syringae* strain B64 isolated from wheat. Stand. Genomic Sci.

[b22] Dudnik A, Dudler R (2013b). High-quality draft genome sequence of *Pseudomonas syringae* pv. *syringae* strain SM, isolated from wheat. Genome Announc.

[b23] Dudnik A, Dudler R (2014a). Genome and transcriptome sequences of *Pseudomonas syringae* pv. *syringae* B301D-R. Genome Announc.

[b24] Dudnik A, Dudler R (2014b). Genomics-based exploration of virulence determinants and host-specific adaptations of *Pseudomonas syringae* strains isolated from grasses. Pathogens.

[b25] Edwards DJ, Holt KE (2013). Beginner’s guide to comparative bacterial genome analysis using next-generation sequence data. Microb. Inform. Exp.

[b26] Feil H, Feil WS, Chain P, Larimer F, DiBartolo G, Copeland A (2005). Comparison of the complete genome sequences of *Pseudomonas syringae* pv. *syringae* B728a and pv. *tomato* DC3000. Proc. Natl. Acad. Sci. USA.

[b27] Gardan L, Shafik H, Belouin S, Broch R, Grimont F, Grimont PAD (1999). DNA relatedness among the pathovars of *Pseudomonas syringae* and description of *Pseudomonas tremae* sp. nov. and *Pseudomonas cannabina* sp. nov. (ex Sutic and Dowson 1959). Int. J. Syst. Bacteriol.

[b28] Gardiner DM, Stiller J, Covarelli L, Lindeberg M, Shivas RG, Manners JM (2013). Genome sequences of *Pseudomonas* spp. isolated from cereal crops. Genome Announc.

[b29] Gonzalez CF, Layher SK, Vidaver AK, Olsen RH (1984). Transfer, mapping, and cloning of *Pseudomonas syringae* pv. *syringae* plasmid pCG131 and assessment of its role in virulence. Phytopathology.

[b30] Grant JR, Stothard P (2008). The CGView Server: a comparative genomics tool for circular genomes. Nucleic Acids Res.

[b31] Greenwald JW, Greenwald CJ, Philmus BJ, Begley TP, Gross DC (2012). RNA-seq analysis reveals that an ECF σ factor, AcsS, regulates achromobactin biosynthesis in *Pseudomonas syringae* pv. *syringae* B728a. PLoS One.

[b32] Grgurina I, Mariotti F, Fogliano V, Gallo M, Scaloni A, Iacobellis NS (2002). A new syringopeptin produced by bean strains of *Pseudomonas syringae* pv. *syringae*. Biochim. Biophys. Acta.

[b33] Gross DC, DeVay JE (1977). Population dynamics and pathogenesis of *Pseudomonas syringae* in maize and cowpea in relation to in vitro production of syringomycin. Phytopathology.

[b34] Gross DC, Cody YS, Proebsting EL, Radamaker GK, Spotts RA (1984). Ecotypes and pathogenicity of ice-nucleation-active *Pseudomonas syringae* isolated from deciduous fruit tree orchards. Phytopathology.

[b35] Gutiérrez-Barranquero JA, Carrión VJ, Murillo J, Arrebola E, Arnold DL, Cazorla FM (2013). A *Pseudomonas syringae* diversity survey reveals a differentiated phylotype of the pathovar *syringae* associated with the mango host and mangotoxin production. Phytopathology.

[b36] Hirano SS, Upper CD (1990). Population biology and epidemiology of *Pseudomonas syringae*. Annu. Rev. Phytopathol.

[b37] Hockett KL, Burch AY, Lindow SE (2013). Thermo-regulation of genes mediating motility and plant interactions in *Pseudomonas syringae*. PLoS One.

[b38] Hockett KL, Renner T, Baltrus DA (2014). 10.1101/011486.

[b39] Holcik M, Iyer VN (1997). Conditionally lethal genes associated with bacterial plasmids. Microbiology.

[b40] Jalan N, Aritua V, Kumar D, Yu FH, Jones JB, Graham JH (2011). Comparative genomic analysis of *Xanthomonas axonopodis* pv. *citrumelo* F1, which causes citrus bacterial spot disease, and related strains provides insights into virulence and host specificity. J. Bacteriol.

[b41] Joardar V, Lindeberg M, Jackson RW, Selengut J, Dodson R, Brinkac LM (2005). Whole-genome sequence analysis of *Pseudomonas syringae* pv. *phaseolicola* 1448A reveals divergence among pathovars in genes involved in virulence and transposition. J. Bacteriol.

[b42] Kang YS, Jelenska J, Cecchini NM, Li YJ, Lee MW, Kovar DR (2014). HopW1 from *Pseudomonas syringae* fisrupts the actin cytoskeleton to promote virulence in Arabidopsis. PLoS Pathog.

[b43] Keane PJ, Kerr A, New PB (1970). Crown gall of stone fruit. II. Identification and nomenclature of *Agrobacterium* isolates. Aust. J. Biol. Sci.

[b44] Kurz M, Burch AY, Seip B, Lindow SE, Gross H (2010). Genome-driven investigation of compatible solute biosynthesis pathways of *Pseudomonas syringae* pv. *syringae* and their contribution to water stress tolerance. Appl. Environ. Microbiol.

[b45] Kvitko BH, Park DH, Velasquez AC, Wei CF, Russell AB, Martin GB (2009). Deletions in the repertoire of *Pseudomonas syringae* pv. *tomato* DC3000 type III secretion effector genes reveal functional overlap among effectors. PLoS Pathog.

[b46] Lan LF, Deng X, Zhou JM, Tang XY (2006). Genome-wide gene expression analysis of *Pseudomonas syringae* pv. *tomato* DC3000 reveals overlapping and distinct pathways regulated by *hrpL* and *hrpRS*. Mol. Plant Microbe Interact.

[b47] Latreille P, Norton S, Goldman BS, Henkhaus J, Miller N, Barbazuk B (2007). Optical mapping as a routine tool for bacterial genome sequence finishing. BMC Genom.

[b48] Li SS, Yu XL, Beattie GA (2013). Glycine betaine catabolism contributes to *Pseudomonas syringae* tolerance to hyperosmotic stress by relieving betaine-mediated suppression of compatible solute synthesis. J. Bacteriol.

[b49] Lin YC, Hu YM, Hsu ST, Tzeng KC, Huang HC (2006). Cloning and characterization of exchangeable effector locus of *Pseudomonas syringae* pv. *averrhoi*, a new pathogen on *Averrhoa carambola*. Plant Pathol. Bull.

[b50] Lindeberg M, Myers CR, Collmer A, Schneider DJ (2008). Roadmap to new virulence determinants in *Pseudomonas syringae*: insights from comparative genomics and genome organization. Mol. Plant Microbe Interact.

[b51] Lindow SE, Arny DC, Upper CD (1978). *Erwinia herbicola*: a bacterial ice nucleus active in increasing frost injury to corn. Phytopathology.

[b52] Loper JE, Hassan KA, Mavrodi DV, Davis EW, Lim CK, Shaffer BT (2012). Comparative genomics of plant-associated *Pseudomonas* spp.: insights into diversity and inheritance of traits involved in multitrophic interactions. PLoS Genet.

[b53] Ma WB, Dong FFT, Stavrinides J, Guttman DS (2006). Type III effector diversification via both pathoadaptation and horizontal transfer in response to a coevolutionary arms race. PLoS Genet.

[b54] Ma ZH, Smith JJ, Zhao YF, Jackson RW, Arnold DL, Murillo J (2007). Phylogenetic analysis of the pPT23A plasmid family of *Pseudomonas syringae*. Appl. Environ. Microbiol.

[b55] Markowitz VM, Mavromatis K, Ivanova NN, Chen IM, Chu K, Kyrpides NC (2009). IMG ER: a system for microbial genome annotation expert review and curation. Bioinformatics.

[b56] Matthews TD, Rabsch W, Maloy S (2011). Chromosomal rearrangements in *Salmonella enterica* serovar Typhi strains isolated from asymptomatic human carriers. MBio.

[b57] Mavromatis K, Chu K, Ivanova N, Hooper SD, Markowitz VM, Kyrpides NC (2009). Gene context analysis in the integrated microbial genomes (IMG) data management system. PLoS One.

[b58] Misas-Villamil JC, Kolodziejek I, Crabill E, Kaschani F, Niessen S, Shindo T (2013). *Pseudomonas syringae* pv. *syringae* uses proteasome inhibitor syringolin A to colonize from wound infection sites. PLoS Pathog.

[b59] Morris CE, Monteil CL, Berge O (2013). The life history of *Pseudomonas syringae*: linking agriculture to earth system processes. Annu. Rev. Phytopathol.

[b60] Nowell RW, Green S, Laue BE, Sharp PM (2014). The extent of genome flux and its role in the differentiation of bacterial lineages. Genome Biol. Evol.

[b61] Nylander JAA (2004). MrModelTest version 2.2.

[b62] O’Brien HE, Desveaux D, Guttman DS (2011a). Next-generation genomics of *Pseudomonas syringae*. Curr. Opin. Microbiol.

[b63] O’Brien HE, Thakur S, Guttman DS (2011b). Evolution of plant pathogenesis in *Pseudomonas syringae*: a genomics perspective. Annu. Rev. Phytopathol.

[b64] Pati A, Ivanova NN, Mikhailova N, Ovchinnikova G, Hooper SD, Lykidis A (2010). GenePRIMP: a gene prediction improvement pipeline for prokaryotic genomes. Nat. Methods.

[b65] Quigley NB, Gross DC (1994). Syringomycin production among strains of *Pseudomonas syringae* pv. *syringae*: conservation of the *syrB* and *syrD* genes and activation of phytotoxin production by plant signal molecules. Mol. Plant Microbe Interact.

[b500] Quinones B, Pujol CJ, Lindow SE (2004). Regulation of AHL production and its contribution to epiphytic fitness in *Pseudomonas syringae*. Mol Plant Microbe Interact.

[b66] Records AR (2011). The type VI secretion system: a multipurpose delivery system with a phage-like machinery. Mol. Plant Microbe Interact.

[b67] Rodriguez-Palenzuela P, Matas IM, Murillo J, López-Solanilla E, Bardaji L, Pérez-Martinez I (2010). Annotation and overview of the *Pseudomonas savastanoi* pv. *savastanoi* NCPPB 3335 draft genome reveals the virulence gene complement of a tumour-inducing pathogen of woody hosts. Environ. Microbiol.

[b68] Ronquist F, Huelsenbeck JP (2003). MrBayes 3: Bayesian phylogenetic inference under mixed models. Bioinformatics.

[b69] Röttig M, Medema MH, Blin K, Weber T, Rausch C, Kohlbacher O (2011). NRPSpredictor2 – a web server for predicting NRPS adenylation domain specificity. Nucleic Acids Res.

[b70] Rutherford K, Parkhill J, Crook J, Horsnell T, Rice P, Rajandream MA (2000). Artemis: sequence visualization and annotation. Bioinformatics.

[b71] Sarkar SF, Guttman DS (2004). Evolution of the core genome of *Pseudomonas syringae*, a highly clonal, endemic plant pathogen. Appl. Environ. Microbiol.

[b72] Sarris PF, Skandalis N, Kokkinidis M, Panopoulos NJ (2010). In silico analysis reveals multiple putative type VI secretion systems and effector proteins in *Pseudomonas syringae* pathovars. Mol. Plant Pathol.

[b73] Schellenberg B, Ramel C, Dudler R (2010). *Pseudomonas syringae* virulence factor syringolin A counteracts stomatal immunity by proteasome inhibition. Mol. Plant Microbe Interact.

[b74] Scholz-Schroeder BK, Hutchison ML, Grgurina I, Gross DC (2001a). The contribution of syringopeptin and syringomycin to virulence of *Pseudomonas syringae* pv. *syringae* strain B301D on the basis of *sypA* and *syrB1* biosynthesis mutant analysis. Mol. Plant Microbe Interact.

[b75] Scholz-Schroeder BK, Soule JD, Lu SE, Grgurina I, Gross DC (2001b). A physical map of the syringomycin and syringopeptin gene clusters localized to an approximately 145-kb DNA region of *Pseudomonas syringae* pv. *syringae* strain B301D. Mol. Plant Microbe Interact.

[b76] Scholz-Schroeder BK, Soule JD, Gross DC (2003). The *sypA*
*sypB* and *sypC* synthetase genes encode twenty-two modules involved in the nonribosomal peptide synthesis of syringopeptin by *Pseudomonas syringae* pv. *syringae* B301D. Mol. Plant Microbe Interact.

[b77] Segre A, Bachmann RC, Ballio A, Bossa F, Grgurina I, Iacobellis NS (1989). The structure of syringomycins A_1_, E and G. FEBS Lett.

[b78] Siguier P, Perochon J, Lestrade L, Mahillon J, Chandler M (2006). ISfinder: the reference centre for bacterial insertion sequences. Nucleic Acids Res.

[b79] Sorensen KN, Kim KH, Takemoto JY (1998). PCR detection of cyclic lipodepsinonapeptide-producing *Pseudomonas syringae* pv. *syringae* and similarity of strains. Appl. Environ. Microbiol.

[b80] Stavrinides J, Guttman DS (2004). Nucleotide sequence and evolution of the five-plasmid complement of the phytopathogen *Pseudomonas syringae* pv. *maculicola* ES4326. J. Bacteriol.

[b81] Stockwell VO, Davis EW, Carey A, Shaffer BT, Mavrodi DV, Hassan KA (2013). pA506, a conjugative plasmid of the plant epiphyte *Pseudomonas fluorescens* A506. Appl. Environ. Microbiol.

[b82] Studholme DJ (2011). Application of high-throughput genome sequencing to intrapathovar variation in *Pseudomonas syringae*. Mol. Plant Pathol.

[b83] Sundin GW, Murillo J (1999). Functional analysis of the *Pseudomonas syringae rulAB* determinant in tolerance to ultraviolet B (290-320 nm) radiation and distribution of *rulAB* among *P. syringae* pathovars. Environ. Microbiol.

[b84] Sundin GW, Mayfield CT, Zhao Y, Gunasekera TS, Foster GL, Ullrich MS (2004). Complete nucleotide sequence and analysis of pPSR1 (72,601 bp), a pPT23A-family plasmid from *Pseudomonas syringae* pv. *syringae* A2. Mol. Genet. Genomics.

[b85] Swofford DL (2002). PAUP, phylogenetic analysis using parsimony (and other methods), version 4.

[b86] Tamura K, Peterson D, Peterson N, Stecher G, Nei M, Kumar S (2011). MEGA5: molecular evolutionary genetics analysis using maximum likelihood, evolutionary distance, and maximum parsimony methods. Mol. Biol. Evol.

[b87] Thakur PB, Vaughn-Diaz VL, Greenwald JW, Gross DC (2013). Characterization of five ECF sigma factors in the genome of *Pseudomonas syringae* pv. *syringae* B728a. PLoS One.

[b88] Vallet-Gely I, Opota O, Boniface A, Novikov A, Lemaitre B (2010). A secondary metabolite acting as a signalling molecule controls *Pseudomonas entomophila* virulence. Cell. Microbiol.

[b89] Vidaver AK (1967). Synthetic and complex media for rapid detection of fluorescence of phytopathogenic pseudomonads: effect of carbon source. Appl. Microbiol.

[b90] Vinatzer B, Gross DC, Lichens-Park A, Kole C, Monteil C (2014). *Pseudomonas syringae* genomics: from comparative genomics of individual crop pathogen strains toward population genomics. Genomics of plant-associated bacteria.

[b91] Vinatzer BA, Teitzel GM, Lee MW, Jelenska J, Hotton S, Fairfax K (2006). The type III effector repertoire of *Pseudomonas syringae* pv. *syringae* B728a and its role in survival and disease on host and non-host plants. Mol. Microbiol.

[b92] Wang XX, Wood TK (2011). Toxin-antitoxin systems influence biofilm and persister cell formation and the general stress response. Appl. Environ. Microbiol.

[b93] Wu X, Monchy S, Taghavi S, Zhu W, Ramos J, van der Lelie D (2011). Comparative genomics and functional analysis of niche-specific adaptation in *Pseudomonas putida*. FEMS Microbiol. Rev.

[b94] Young JM (2010). Taxonomy of *Pseudomonas syringae*. J. Plant Pathol.

[b95] Yu XL, Lund SP, Scott RA, Greenwald JW, Records AH, Nettleton D (2013). Transcriptional responses of *Pseudomonas syringae* to growth in epiphytic versus apoplastic leaf sites. Proc. Natl. Acad. Sci. USA.

[b96] Yu X, Lund SP, Greenwald JW, Records AH, Scott RA, Nettleton D (2014). Transcriptional analysis of the global regulatory networks active in *Pseudomonas syringae* during leaf colonization. MBio.

[b97] Zhao YF, Ma ZH, Sundin GW (2005). Comparative genomic analysis of the pPT23A plasmid family of *Pseudomonas syringae*. J. Bacteriol.

[b98] Zhou Y, Tao J, Yu H, Ni JJ, Zeng LB, Teng QH (2012). Hcp family proteins secreted via the type VI secretion system coordinately regulate *Escherichia coli* K1 interaction with human brain microvascular endothelial cells. Infect. Immun.

