# Supplementary figures and images for: Comparative genomics of Pseudomonas syringae pv. syringae strains B301D and HS191 and insights into intrapathovar traits associated with plant pathogenesis
Source: Microbiologyopen. 2015 May 4;4(4):553–73. doi: 10.1002/mbo3.261 (PMC4554452; doi:10.1002/mbo3.261)

**B301D chromosome**

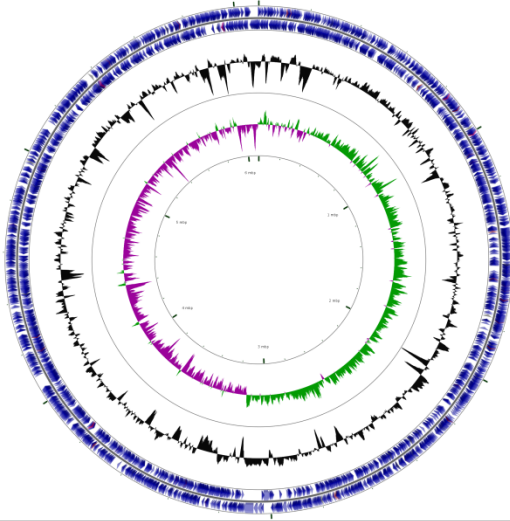

**HS191 chromosome and pCG131 plasmid**

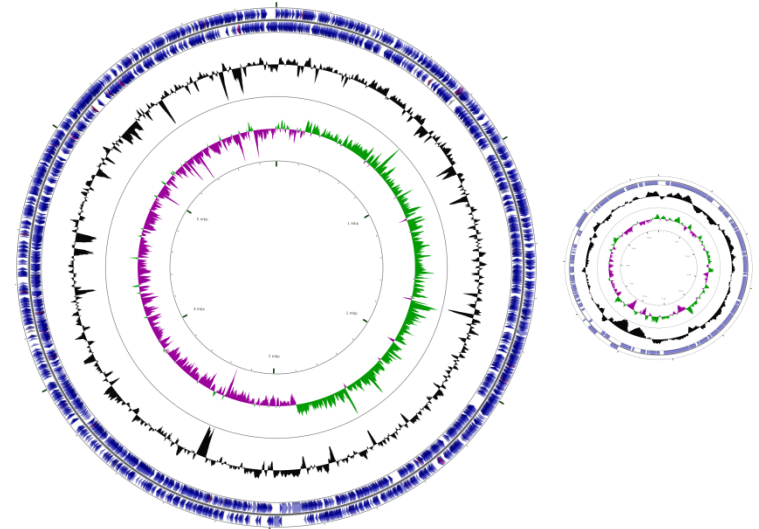

Supplement: Supplementary file 1 [file mbo30004-0553-sd1.pdf]

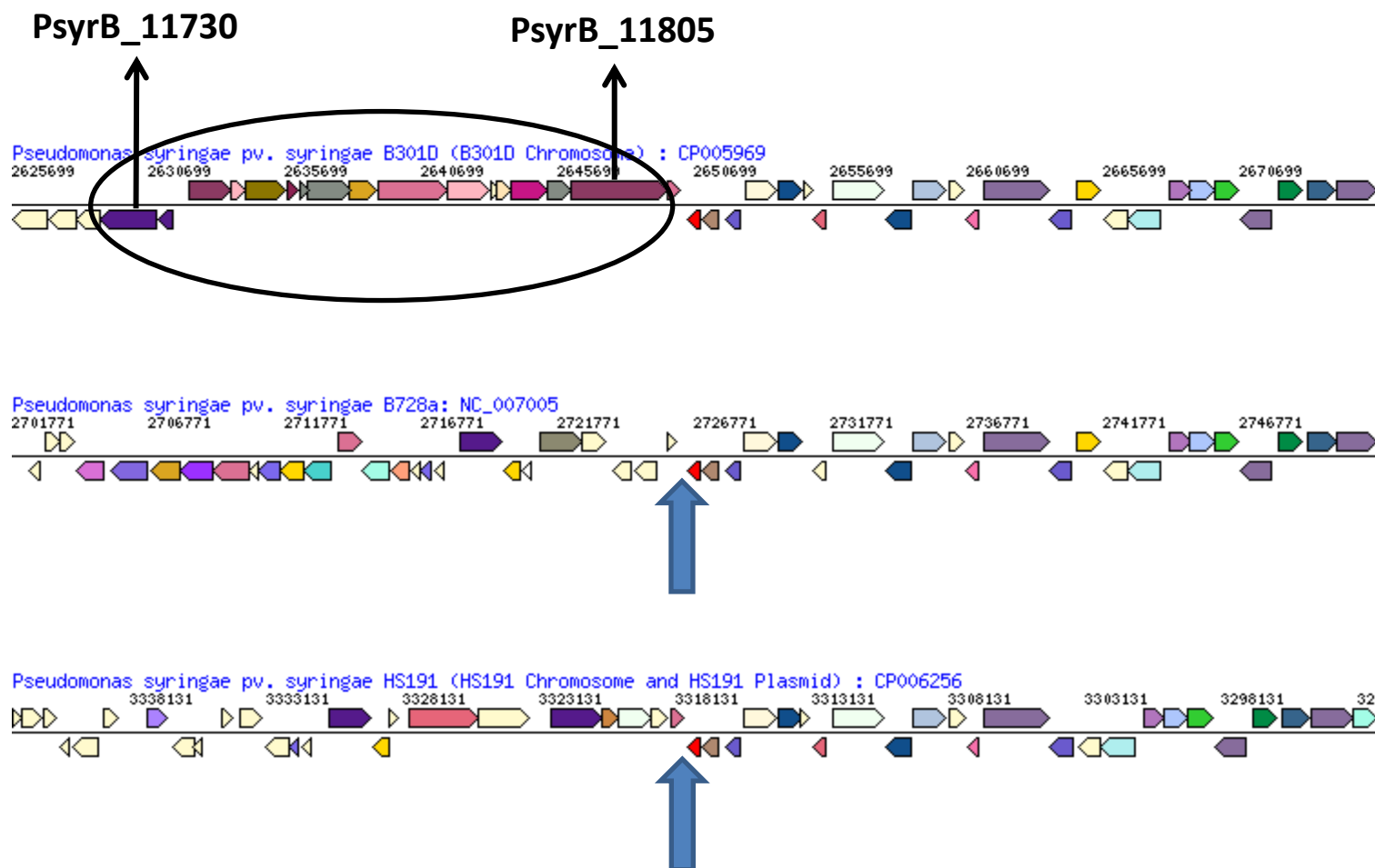

Supplement: Supplementary file 2 [file mbo30004-0553-sd2.pdf]

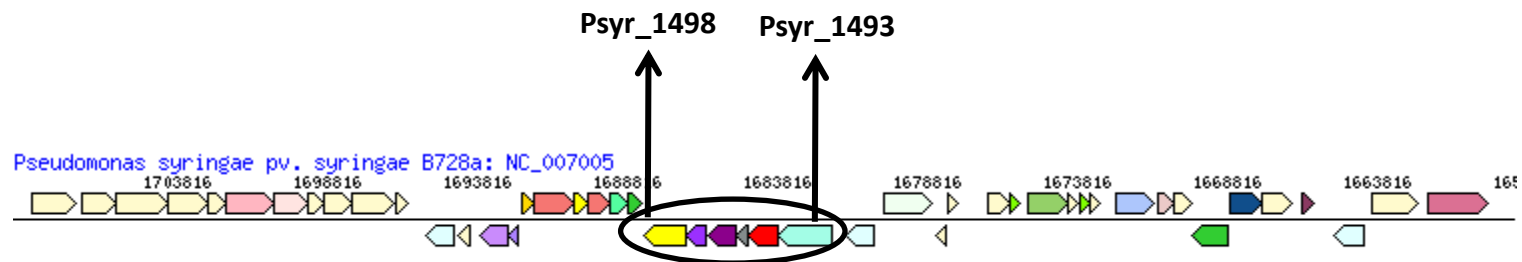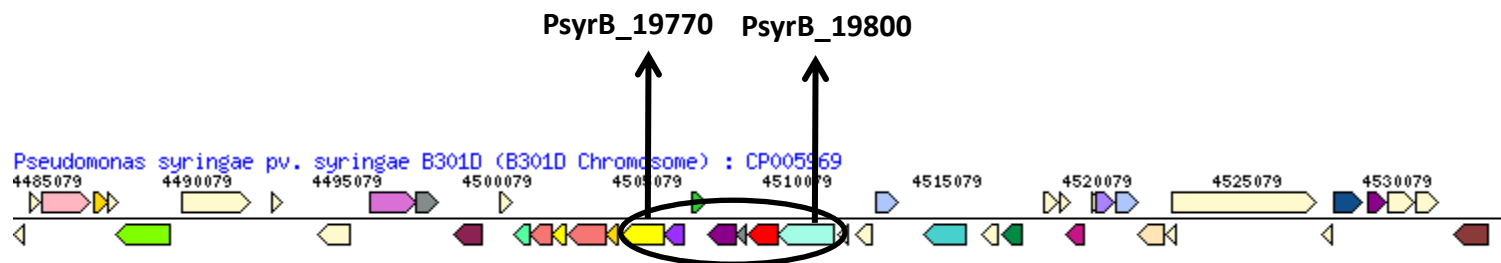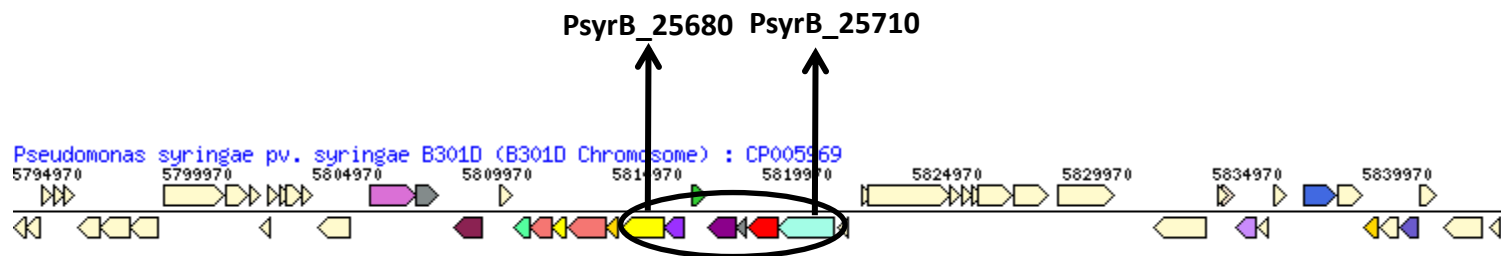

Supplement: Supplementary file 3 [file mbo30004-0553-sd3.pdf]

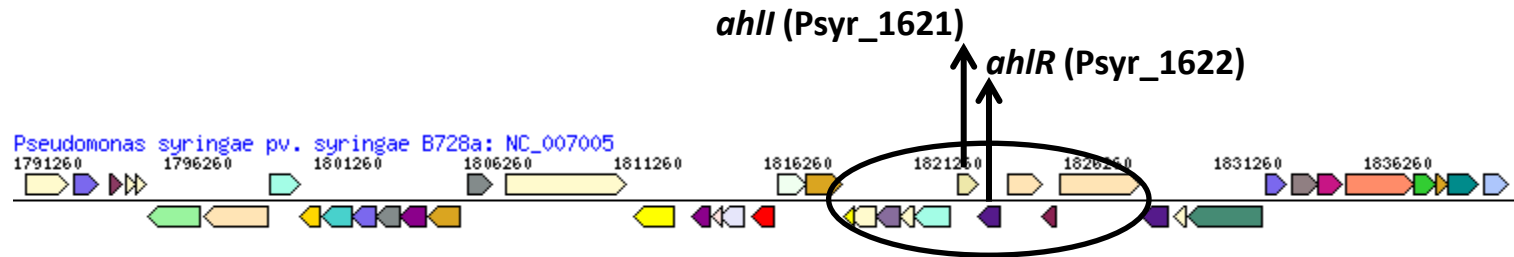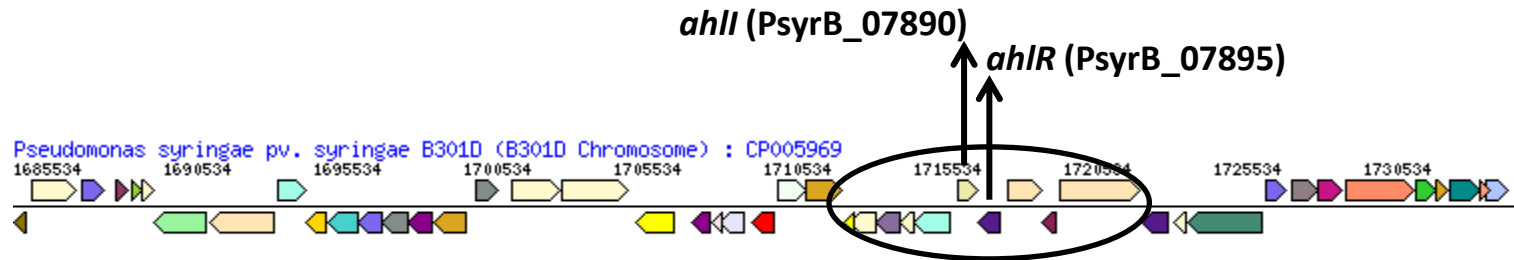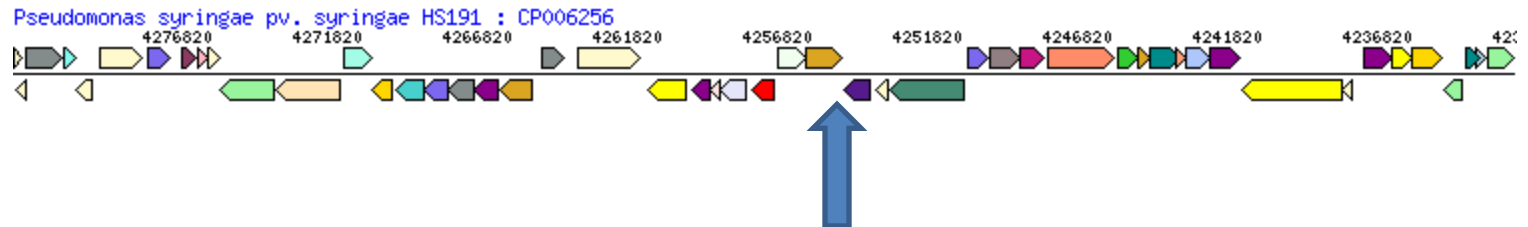

Supplement: Supplementary file 4 [file mbo30004-0553-sd4.pdf]

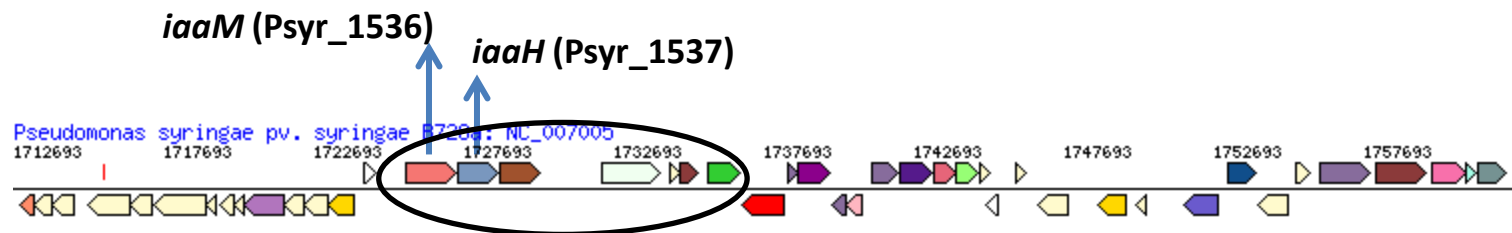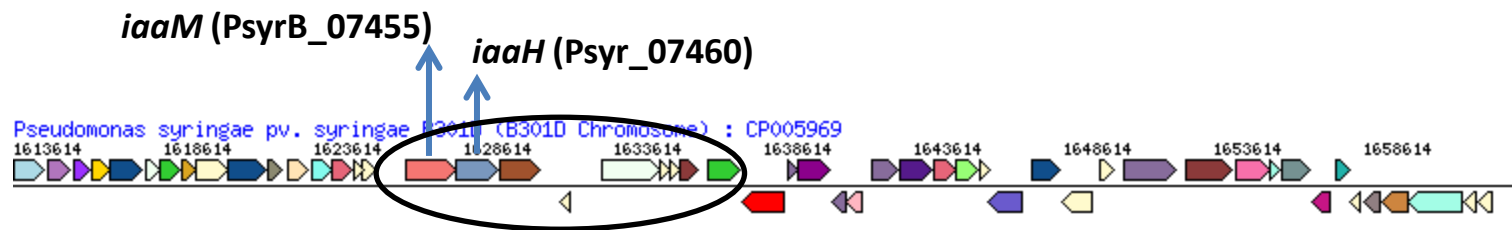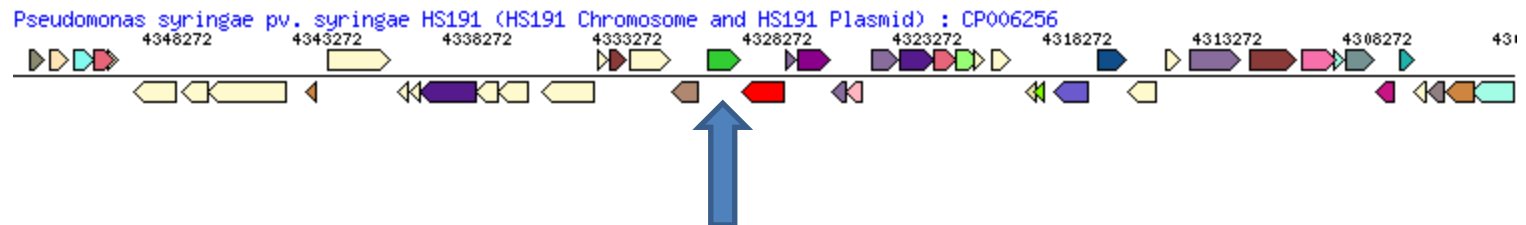

Supplement: Supplementary file 5 [file mbo30004-0553-sd5.pdf]

## Psyr\_2195 - 2205

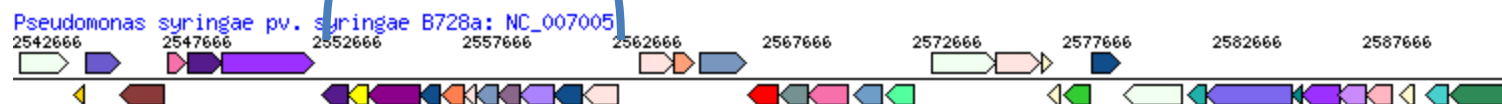

## PsyrB\_10955 - 11005

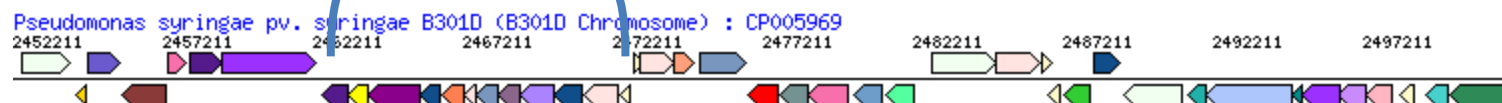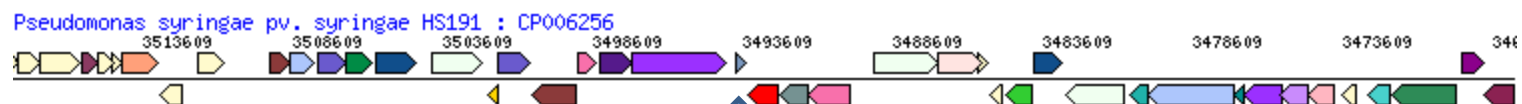

Supplement: Supplementary file 6 [file mbo30004-0553-sd6.pdf]

A

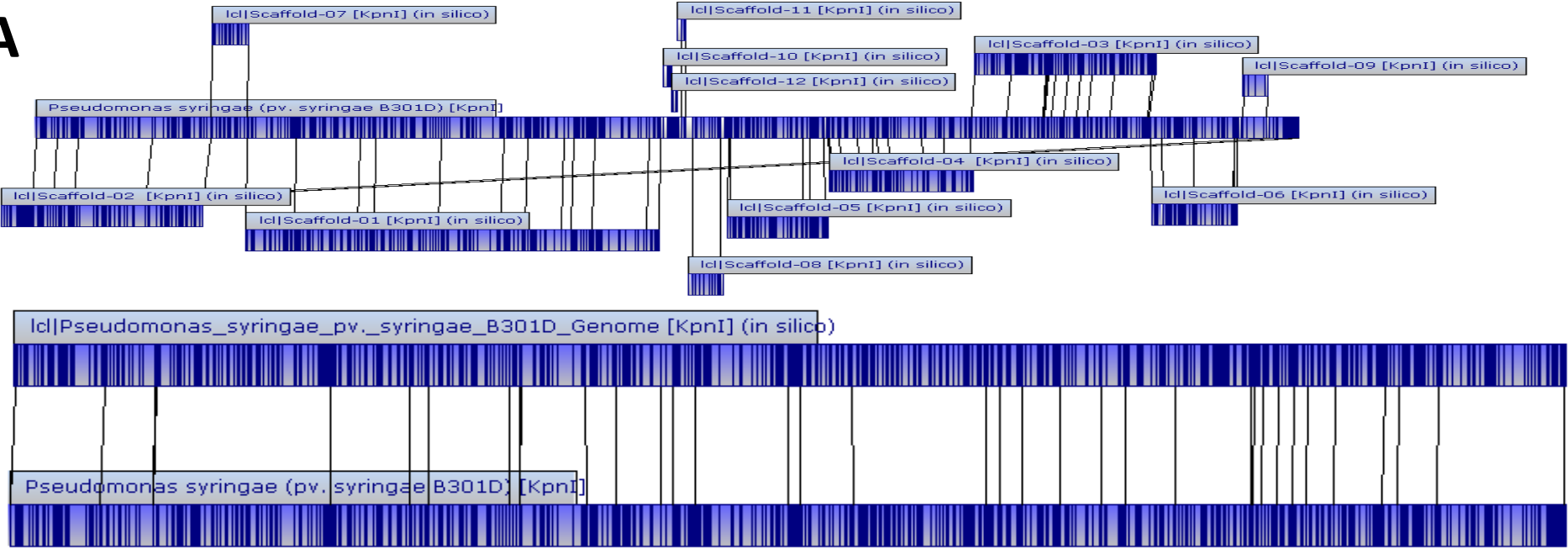

B

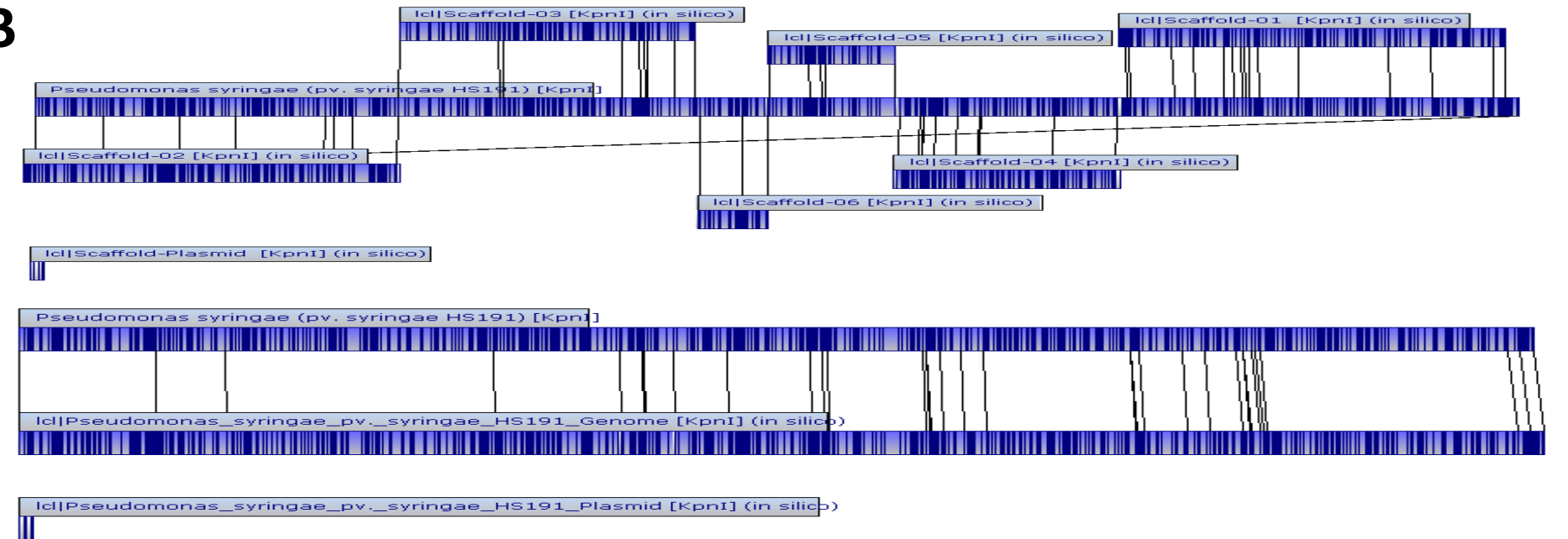

Supplement: Supplementary file 7 [file mbo30004-0553-sd7.pdf]
