# Supplementary material for: Comparative genomics of Pseudomonas syringae pv. syringae strains B301D and HS191 and insights into intrapathovar traits associated with plant pathogenesis
Source: Microbiologyopen. 2015 May 4;4(4):553–73. doi: 10.1002/mbo3.261 (PMC4554452; doi:10.1002/mbo3.261)
Supplement: Supplementary file 8 [file mbo30004-0553-sd8.docx]

**Table S1.** IS element comparison for the *P. syringae* pv. *syringae* strains B728a, B301D and HS191.

| **Family/**  **Group** | **Elements** | **Origin** | **B728a**  **locus_tag (Psyr_)** | **B301D**  **locus_tag (PsyrB_)** | **HS191**  **locus_tag (PsyrH_)** |
| --- | --- | --- | --- | --- | --- |
| IS3 | ISPsy8 | *P. syringae* pv. tomato DC3000 | 1976-1975  2653-2654  3316-3317  4859-4860 | 11440-11430 | -- |
| IS3 | ISPsy9 | *P. syringae* pv. tomato DC3000 | 0274-0273  1177-1176  4617-4616  4679-4680  0593-0592 | 01645-01650  24465-24460 | 16715-16720 |
| IS3/IS3 | ISPsy28 | *P. syringae* | 0106-0105  3811-3810 | 02225-02230  24420-24425 | -- |
| IS5/IS5 | IS2000 | *P. aeruginosa* JES | -- | -- | 00475 |
| IS5/IS5 | ISPsy2 | *P. syringae* pv. *eriobotryae* | 0096 | -- | 19725-19730 |
| IS5/IS427 | ISPs1 | *P. syringae* pv. *syringae* plasmid pPSR1 | -- | -- | 20090 |
| IS66 | ISPpu19 | *P. putida* MT53 plasmid pWW53 | 0747-0743 | 19685-19690 | -- |
| IS66 | ISPsy5 | *P. syringae* pv. tomato DC3000 | 1223-1221 | -- | -- |
| IS630 | ISPsy25 | *P. syringae* pv. *phaseolicola* | -- | 19620-19615 | -- |
| IS1182 | ISPsy27 | *P. syringae* pv. *syringae* B728a | 0095 | 10010 | -- |

-- indicates absence

**Table S2.** Prophage finder of *P. syringae* pv. *syringae* strains B728a, B301D and HS191.

| **Chromosome** | **Prophages** | | | **Tailocin** |
| --- | --- | --- | --- | --- |
|  | Prophage 1 Type | Prophage 2 Type | Prophage 3 Type |  |
| B728a | Psyr_2761- 2821  48.0 kb (60 genes) | -- | -- | Psyr_4578- 4611  29.1 kb (34 genes) |
| B301D | PsyrB_13920- 14250  47.3 kb (67 genes) | PsyrB_25460- 25640  28.7 kb (37 genes) | -- | PsyrB_23745- 23915  28.1 kb (35 genes) |
| HS191 | -- | -- | PsyrH_15245- 15480  34.2 kb (47 genes) | PsyrH_23505- 23680  29.0 kb (36 genes) |

-- indicates absence
